# The effect of TG2-inhibitory monoclonal antibody zampilimab on tissue fibrosis in human *in vitro* and primate *in vivo* models of chronic kidney disease

**DOI:** 10.1371/journal.pone.0298864

**Published:** 2024-05-16

**Authors:** Linghong Huang, Helene Bon, Mabrouka Maamra, Toby Holmes, John Atkinson, Katharine Cain, Jeff Kennedy, Catherine Kettleborough, David Matthews, Breda Twomey, Jia Ni, Zhizhan Song, Philip F. Watson, Timothy S. Johnson

**Affiliations:** 1 Immunology Therapeutic Area, UCB Pharma, Slough, United Kingdom; 2 UCB Pharma, Slough, United Kingdom; 3 Department of Oncology and Metabolism, Medical School, University of Sheffield, Sheffield, United Kingdom; 4 Drug Discovery Biology, LifeArc, Stevenage, United Kingdom; 5 Immunology and Ophthalmology, Mogrify Ltd, Cambridge, United Kingdom; 6 Research and Development, Prisys Biotechnologies, Shanghai, China; Max Delbruck Centrum fur Molekulare Medizin Berlin Buch, GERMANY

## Abstract

Fibrotic remodeling is the primary driver of functional loss in chronic kidney disease, with no specific anti-fibrotic agent available for clinical use. Transglutaminase 2 (TG2), a wound response enzyme that irreversibly crosslinks extracellular matrix proteins causing dysregulation of extracellular matrix turnover, is a well-characterized anti-fibrotic target in the kidney. We describe the humanization and characterization of two anti-TG2 monoclonal antibodies (zampilimab [hDC1/UCB7858] and BB7) that inhibit crosslinking by TG2 in human *in vitro* and rabbit/cynomolgus monkey i*n vivo* models of chronic kidney disease. Determination of zampilimab half-maximal inhibitory concentration (IC_50_) against recombinant human TG2 was undertaken using the KxD assay and determination of dissociation constant (K_d_) by surface plasmon resonance. Efficacy *in vitro* was established using a primary human renal epithelial cell model of tubulointerstitial fibrosis, to assess mature deposited extracellular matrix proteins. Proof of concept *in vivo* used a cynomolgus monkey unilateral ureteral obstruction model of chronic kidney disease. Zampilimab inhibited TG2 crosslinking transamidation activity with an IC_50_ of 0.25 nM and K_d_ of <50 pM. In cell culture, zampilimab inhibited extracellular TG2 activity (IC_50_ 119 nM) and dramatically reduced transforming growth factor-β1-driven accumulation of multiple extracellular matrix proteins including collagens I, III, IV, V, and fibronectin. Intravenous administration of BB7 in rabbits resulted in a 68% reduction in fibrotic index at Day 25 post-unilateral ureteral obstruction. Weekly intravenous administration of zampilimab in cynomolgus monkeys with unilateral ureteral obstruction reduced fibrosis at 4 weeks by >50%, with no safety signals. Our data support the clinical investigation of zampilimab for the treatment of kidney fibrosis.

## Introduction

Fibrotic tissue remodeling is characterized by excessive accumulation of extracellular matrix (ECM) components and uncontrolled proliferation of myofibroblasts, leading to epithelial death, loss of organ architecture, and functional decline. Fibrosis typically is a consequence of chronic disease such as hypertension, diabetes, and long-term inflammatory conditions [1, 2]. Approximately 45% of deaths in the United States are attributable to fibrotic diseases [3]. Due to the associated morbidity and mortality, a wide variety of potential therapeutic targets have been identified to prevent or reverse fibrosis [4]. However, there remains a dearth of effective therapeutic options available in the clinic [4], with pirfenidone and nintedanib the only licensed therapies and only in idiopathic pulmonary fibrosis/interstitial lung diseases [5]. Neither agent is fully effective, and both are associated with gastrointestinal side effects [4, 5].

Transglutaminase 2 (TG2), a calcium-dependent, crosslinking enzyme that catalyzes the formation of ε-(ɣ-glutamyl)-lysine isopeptide bonds between adjacent peptides in the ECM and elsewhere, is heavily implicated in fibrosis [6, 7]. TG2 is trafficked to the extracellular environment following tissue injury [7, 8], and plays a significant role in ECM homeostasis [6, 7]. Elevated extracellular TG2 levels accelerate ECM deposition as ε-(ɣ-glutamyl)-lysine crosslinking short circuits normal assembly pathways. The incorporation of these bonds reduces breakdown and clearance of ECM proteins by matrix metalloproteinases, moving the ECM homeostatic balance in favor of accumulation. Further TG2 release can locally cross-link large latent transforming growth factor-β (TGF-β) into the ECM, initiating its activation [9] and pro-fibrotic effects [10]. Chronic activation of these processes can switch the beneficial wound response actions of TG2 to a pathological scarring and fibrosis [11]. Human disease expression data [12, 13] and studies using knock-out (KO) animals [11, 14], together with studies on small-molecule inhibitors [15, 16] and small interfering ribonucleic acid (siRNA) [12, 17], have suggested TG2 as a favorable therapeutic target in kidneys [18], lungs [19], liver [20], and heart [15]; this concept is perhaps best supported by data in chronic kidney disease (CKD) [18, 21, 22].

TG2-specific inhibitors have proven difficult to develop. The TG catalytic core is highly conserved across the eight-member TG family, some of which are key to human physiology [6], such as Factor Xllla (clotting), TG1, and TG3 (terminal differentiation of the keratinocyte). The challenge has therefore been to generate selective TG2 inhibitors, avoiding inhibition of other TGs.

To address this, using immunization of mice with TG2 domain fragments, we previously identified 13 high-affinity inhibitory monoclonal antibodies specific to TG2, mapped to four previously unreported inhibitory epitopes within the catalytic core [23]. Here we describe the humanization and characterization of the two most effective antibodies from the previous study [23]. Using CKD as an exemplar of fibrotic diseases, we evaluated the lead antibody (DC1) in an *in vitro* primary human cell model of fibrosis. A rabbitized version of the backup antibody BB7 (rbBB7), which has some species cross-reactivity, was then tested in a rabbit model of CKD before evaluating the humanized and human-specific zampilimab in a cynomolgus monkey model of tubulointerstitial fibrosis.

## Materials and methods

### Antibody selection, humanization, and characterization

#### Determining the sequence of the antibody variable heavy chain (VH) and variable light chain (VL) regions

RNA was extracted from hybridomas expressing antibodies BB7 and DC1, reverse transcribed and amplified by polymerase chain reaction (PCR) using degenerate PCR primers [24]. Amino acid sequences for the VH regions and VL regions are shown in S1.1 and S1.2 Tables of S1 File.

#### Design of humanized antibodies

The protein sequences of human and mouse immunoglobulins from the International Immunogenetics Database 2009 [25] and the Kabat Database Release 5 of Sequences of Proteins of Immunological Interest [26] were used to compile a database of human immunoglobulin sequences in Kabat alignment containing 10,906 VH and 2912 variable kappa light chain (VK) sequences. A homology model of the mouse antibody variable regions was calculated using the Modeller program [27] run in automatic mode. The atomic coordinates of 1 MQK.pdb, 3 LIZ.pdb, and 1 MQK.pdb were the highest identity sequence templates for the Interface, VL, and VH, respectively, as determined by Basic Local Alignment Search Tool (BLAST) analysis of the Accelrys antibody Protein Data Bank (pdb) structures database. These templates were used to generate 20 initial models, the best of which was refined by modeling each complementarity-determining region (CDR) loop with its three best loop templates. To select the most appropriate human framework sequence, the sequence analysis program, gibsSR, was used to interrogate the human VH and VK databases with the BB7 VH constant region © and VKc and the DC1 VHc and VKc protein sequences using various selection criteria. Framework (FW) residues within 5Å of a CDR residue (Kabat definition) in the homology model of mouse antibody were identified and designated as the “5Å Proximity” residues.

AF062260 was chosen as the framework on which to base the humanized heavy chain versions. S1.1 Table in S1 File shows the alignment and residue identity of AF062260 to murine antibodies. AF193851 was chosen as the framework on which to base the humanized VK constructs. The alignment and residue identity to the murine antibodies are shown in S1.2 Table of S1 File. To design the final humanized variable regions for BB7 and DC1, the “5Å Proximity” residues in the human framework were back mutated to their equivalent in the murine BB7 or DC1 variable region. The sequence alignments of the final humanized versions of BB7 (hBB7) and DC1 (hDC1) VH regions are shown in S1.3 Table of S1 File, and the sequence alignments of the final humanized versions of BB7 and DC1 VK regions are shown in S1.4 Table of S1 File. hDC1 was developed as the lead therapeutic candidate and is also known as zampilimab (UCB7858).

#### Generation of humanized antibodies

The DNA sequences of the humanized antibodies hDC1 and hBB7 variable regions and leader sequences were optimized and synthesized by GeneArt^®^ (Thermo Fisher Scientific, UK). The sequences were then inserted via ligation into vectors designed to express humanized kappa light and heavy chains in mammalian cells, as previously described [28, 29].

Heavy and kappa light chain vectors for each humanized antibody were co-transfected into Expi293 cells (Thermo Fisher Scientific) in accordance with manufacturer’s instructions for 7–10 days. Antibody was purified from tissue culture supernatant by Protein A or G affinity chromatography. Quality control for each batch of antibody included sodium dodecyl sulphate–polyacrylamide gel electrophoresis analysis and endotoxin testing by Endosafe Portable Testing System (PTS) (Charles River Laboratories, UK). All batches were required to have a purity of >99% and <0.05 EU/mL. Detailed protocols have been previously described [24].

To reduce the risk of xenoreactive immune responses that could arise from the use of a chimeric (non-human variable region) or non-human antibody when administered in patients, both antibodies were fully humanized by replacing the constant regions and by CDR grafting [24]. The same human variable regions were used to humanize both antibodies. Humanized antibodies, hDC1 and hBB7, were generated with no defined functional difference in both immunoglobulin (Ig)G1 and IgG4P formats. To facilitate initial proof-of-principle *in vivo* studies, as BB7 had the ability to inhibit rabbit TG2, hBB7 was subsequently “rabbitized” as rbBB7. The framework incorporating the BB7 CDRs was altered to include more rabbit-like residues in their VK and VH regions.

#### Determination of TG2 antibody dissociation constant (K_d_) using surface plasmon resonance

Surface plasmon resonance was performed using a BIAcore™ T200 instrument (GE Healthcare, UK). Affinipure F(ab’)2 fragment goat anti-human IgG, specific for Fcγ fragment (Jackson ImmunoResearch, UK; 109-006-098), was immobilized on a CM5 Sensor Chip (GE Healthcare) via amine coupling to a capture level of ~3000–5000 response units (RUs) enabling antibody capture. A running buffer of 10 mM HEPES pH 7.4, 0.15 M NaCl, 0.005% Surfactant P20 (GE Healthcare), and 2 mM CaCl_2_, was used with a flow rate of 10 μL/min. A 10 μL/min injection of test antibody at 0.1 μg/mL or 1 μg/mL was made to achieve capture levels of ~100–200 RU by the goat anti-human IgG, Fcγ fragment-specific ligand.

TG2 (human, cynomolgus, and rabbit TG2 produced internally by UCB Pharma, UK) was titrated over the captured test antibody at the required concentrations (ranging from 100 nM to 6.25 nM), diluted in running buffer, at a flow rate of 30 μL/min. The TG2 was injected for 600 seconds of association time. Buffer was then injected for up to 3200 seconds for the dissociation phase. The surface was regenerated by three 30- or two 60-second injections of 40 mM HCl, followed by a 60-second injection of 5 mM NaOH at a flow rate of 10 μL/min. Background-subtracted binding curves were analyzed using BIAevaluation software (Version 3.2; GE Healthcare), following standard procedures. K_d_ was determined from the 1:1 fitting algorithm.

#### Determination of TG2 antibody half-maximal inhibitory concentration (IC_50_) using the KxD TG activity assay

A 5 μL antibody dilution was pre-incubated with 10 μL recombinant TG2 for 1 h at room temperature (RT) in a black, low-volume, low-binding, 384-well plate. A 10 μL mix of N,N-dimethyl casein (NMC) and KxD (N-Boc-Lys-NH-CH(2)-CH(2)-NH-dansyl; Zedira, Germany) was subsequently added, and the reaction mix was incubated for a further 3 h at RT before measuring the fluorescent signal on an EnVision™ (Ex: 320 nm, Em: 510 nm; PerkinElmer, UK) or LJL Analyst (Ex: 330 nm, Em: 485 nm; LJL Biosystems, Inc., USA) machine. Final assay concentrations were human and cynomolgus TG2: 2 nM; rabbit TG2: 1 nM; rat TG2: 1 nM; murine TG2: 1 nM; NMC: 10 μM; KxD: 20 μM. The buffer was: 25 mM HEPES pH 7.4, 250 mM NaCl, 2 mM MgCl_2_, 5 mM CaCl_2_, 2 mM dithiothreitol (DTT), 0.05% Pluronic F127.

The maximum signal was generated in the absence of antibody, and the minimum signal was generated by omitting TG2. Antibody effect was expressed as percentage inhibition of the maximum signal (after subtraction of the minimum signal from both). The percentage inhibition at the different antibody concentrations was used to calculate the IC_50_ using a 4-parameter logistic curve fit. The IC_50_ value was only reported where there were at least two data points above or below this value.

### Measurement of TG2 extracellular activity in cell culture

Human renal proximal tubular epithelial cells (RPTEC; Innoprot, Spain) or rabbit RPTEC and renal fibroblast co-cultures were seeded at 5000 cells per well in a 96-well plate (Becton & Dickinson, UK) in the presence of 0.3–1000 nM of hDC1, rbBB7 or control antibody, and 30 ng/mL TGF-β1 (Peprotech, UK), in a final volume of 200 μL renal epithelial cell basal medium with 0.5% fetal calf serum (FCS), supplements (ATCC-PCS-400-030; ATCC, USA), and 500 μM calcium. After 7 days incubation at 37°C in 5% CO_2_, medium was removed and 150 μL of 50 μM N-(biotinyl) cadaverine (Zedira) were subsequently added in serum-free renal epithelial cell basal medium and incubated for 90 min at 37°C. The reaction was then stopped with 10 mM ethylenediaminetetraacetic acid (EDTA) followed by a cell lysis step with 100 μL 0.25 M NH_4_OH/25 mM Tris for 10 min at RT, phosphate-buffered saline (PBS) washes, and 30 min incubation with 5% bovine serum albumin (BSA) in PBS as blocking buffer. TG-incorporated (biotinyl) cadaverine was revealed with 1:2000 streptavidin–horseradish peroxidase (HRP) conjugate (RPN1051V, GE Healthcare) for 1 h at RT, followed by PBS washes, and detection with 15 min incubation of K-Blue 3,3’5,5’ tetramethylbenzidine (TMB) substrate (331177; Neogen, UK), and stopped with 5 min incubation with Red Stop buffer (301476, Neogen) at RT, before reading on the Synergy2 Multi-Mode reader (650 nm; BioTek, USA).

### Preparation of rabbit primary cells

Renal tubular epithelial cells and fibroblasts were isolated from New Zealand White rabbits. The animals were anesthetized and terminated using Euthatal (Merial Animal Health Ltd., UK; 200 mg/mL): 1 mL Euthatal was diluted in 3 mL PBS and injected via the ear vein to render the animal unconscious (normally occurring within 1 min), followed by 5 mL of neat Euthatal, once the animal had lost pedal reflexes, to complete the termination. Absence of reflexes and cessation of circulation were used to confirm death. The kidneys were removed and immediately washed in ice-cold PBS containing penicillin-streptomycin and fungicide. The cortices of the kidneys (containing the tubules and glomeruli) were isolated and minced into 1 mm^3^ pieces and incubated with 1 mg/mL collagenase (C1639, Sigma-Aldrich) at 37°C for 30 min. This was pulped and then pushed through successive sieves (300–80 μm) to isolate the tubules. The tubules were retained on the 180 μm sieve and the glomeruli washed off into the waste. The tubular fractions were then grown in renal epithelial cell basal medium (ATCC) and fibroblast growth medium (Lonza, Switzerland) to selectively isolate populations of tubular epithelial cells and fibroblasts.

### Quantitative assessment of ECM accumulation

#### Individual ECM proteins by multiplex immunofluorescence (human)

Human primary RPTECs (InnoProt) were seeded at 2000 cells per well in a black clear-bottomed 384-well plate (Greiner Bio-One, Germany) in renal epithelial cell basal medium + 0.5% FCS and supplements (ATCC-PCS-400-040; ATCC). Cells were stimulated with 30 ng/mL TGF-β1 in media supplemented with 500 μM calcium in the presence of either zampilimab or control antibody (A33 IgG4, UCB). After 7 days incubation at 37°C in 5% CO_2_, cells were washed in PBS and lysed with 20 μL 0.25 M NH_4_OH/25 mM Tris for 15 min at 37°C. ECM remaining on the plate was washed three times in PBS, fixed in 40 μL 100% methanol for 30 min at –20°C, and washed three times in PBS before staining. Individual ECM proteins were identified using multiplex immunofluorescence as previously described [30, 31]. In brief, anti-collagen I (Millipore, UK; AB745), anti-collagen III (Millipore, AB747), anti-collagen IV (eBiosciences, 14-9871-82), anti-collagen V (Abcam, UK; ab7046), and anti-fibronectin (eBiosciences™, Thermo Fisher Scientific; 53-9869-82) antibodies were all applied at 1:100 dilution to assess ECM within the cell model. Plates were scanned on the Cellomics ArrayScan HCS reader (Thermo Fisher Scientific) using a three- or four-channel protocol under the “Cellomics CellHealth” profiling bioapplication and a 10x objective (x1 camera) with 2x2 binning (1104x1104 pixels/field). The protocol used a “low-pass filter” background correction method with a value of 71 for all channels, and a fixed threshold object identification method with thresholding.

#### Total ECM by isotopic labeling (human and rabbit)

Primary human RPTECs (5000 cells; InnoProt) or a rabbit primary co-culture of 2000 RPTECs and 2000 renal fibroblasts was grown in 200 μL of renal epithelial cell basal medium (ATCC) containing 1 mmol/L calcium chloride and 0.75 μCi/mL [^14^C]-L-amino acid mixture (PerkinElmer) in a 96-well cytostar scintillation plate (PerkinElmer). A pro-fibrotic response was induced by the addition of 30 ng/mL TGF-β1 (Peprotech) for human cells and 10 ng/mL for rabbit cells. Between 0.75 and 400 μg/mL of zampilimab or rbBB7 antibodies were added per well. After 7 days incubation at 37°C in 5% CO_2_, medium was removed and cells were washed twice with PBS and lysed with 50 μL of 0.25 mol/L NH_4_OH in 50 mmol/L Tris for 10 min at RT. Plates were washed six times and the incorporation of [^14^C]-L-amino acids into the mature ECM was measured using TriLux 1450 with ParaLux Count in ‘High Efficiency’ mode (PerkinElmer).

#### Total ECM by flamingo pink staining (human and rabbit)

Staining for total mature ECM was performed using flamingo pink staining as previously described [32]. Co-cultured rabbit RPTECs and fibroblasts were seeded and cultured for ~7 days with the appropriate stimulus in black/clear 96-well plates or 384-well plates suitable for cellomics ArrayScan imaging. Cells were then removed with 0.5 M NH_4_OH in 50 mmol/L Tris for 10 min at RT and the matrix fixed with methanol at –20°C for 30 min, washed with 150 μL Flowfusor water (x3), and stained with 1x flamingo stain (Bio-Rad, UK) in the dark overnight. This was then washed and 4’,6-diamindino-2-phenylindole, dihydrochloride (DAPI) added to assist with image focusing, and the staining was analyzed on the ArrayScan (Thermo Fisher Scientific).

### Scratch wound assay

Scratch wound assays are often used to assess the potential of anti-fibrotic approaches [33]. WI-38 cells (normal human lung fibroblasts; ATCC) were plated in an Incucyte^®^ Imagelock 96-well Plate (Sartorius, Germany) at 2x10^4^ cells per well in minimum essential medium–alpha modification (αMEM; Thermo Fisher Scientific) with 10% FCS and grown overnight to >97% confluence. Cells were washed twice with αMEM without serum and a scratch wound was generated using the Incucyte^®^ Woundmaker Tool (Sartorius) according to the manufacturer’s protocol. The medium was removed and replaced with 95 μL serum-free medium per well. Controls and test antibodies were added to the wells. The plate was placed in an Incucyte^®^ Scratch Wound Analysis Software Module and the closure of the wound was analyzed using the scratch wound protocol.

Cytochalasin D was used as an assay control at 0.1 μM. A peptide non-reversible TG inhibitor, ZDON (Zedira), was tested at 10 μM and 100 μM to demonstrate a dose-dependent inhibitory effect of TG2. Antibodies zampilimab and hBB7 (produced by LifeArc, UK) were tested on at least three occasions at various concentrations.

Similar assays were also carried out using A549 cells (human alveolar basal epithelial cells; ATCC, US). Cells were plated as above at 2x10^4^ cells per well in Dulbecco’s modified Eagle’s medium with 10% FCS and grown overnight to >97% confluence. Scratches were generated and cells were treated as detailed above.

### Unilateral ureteral obstruction (UUO) model of CKD

All animal work was completed in accordance with the Institutional Animal Care and use Committee (IACUC) guidelines and the Guide for the Care and Use of Laboratory Animals. For both UUO models, no blinding was used to deliver the relevant treatment to the animals; however, all subsequent analysis of tissue was blinded with only the lead scientist at each site aware of the experimental groups, which were uncoded once all analysis was complete.

#### Rabbit

The UUO was performed in adult female New Zealand White rabbits (Highgate Farm, UK) at 2.5–2.9 kg body weight at the University of Sheffield under UK Home Office Project License PBE09C70E (valid December 13, 2016–2021 under Dr Philip Watson and prior to that, Licence 40/3660 under Dr John Haylor). Anesthesia was initiated with pre-med subcutaneous (SC) injections of ketamine (10 mg/kg Ketaset^®^; Covetrus, UK) mixed with xylazine (1 mg/kg Rompun^®^; Covetrus). Once sedated, a 23-gauge butterfly cannula was inserted into the ear vein and induction of anesthesia commenced by slowly introducing 2–3 mL of propofol (10 mg/kg). Maintenance of anesthesia was performed initially using 3% isoflurane by inhalation with oxygen (1 L/min), slowly increasing up to 4.5–5% isoflurane over 10 min using a Fluovac system (Harvard Apparatus, USA). Close observation was maintained during this period as rabbits occasionally stop breathing during administration of isoflurane at this higher dose. If this occurred, the level of isoflurane was temporarily decreased, and respiration re-established by manual palpitation. Surgery commenced once anesthesia was established at 4%, with regular normal respiration.

The lower abdomen was shaved up to the diaphragm exposing the midline abdomen, the abdomen was washed down with an iodine-based surgical scrub, and SC lidocaine was administered along the length of the proposed incision site. A midline laparotomy was performed exposing the peritoneal cavity and intestines, which were positioned on sterile drapes to provide a clear view of the operating field. Adipose tissue was cleared to allow location of the left ureter, and this was tied off with 6–0 Prolene non-absorbable monofilament suture passed through the fat and under the ureter to give total obstruction. The intestines were carefully arranged back in place and the laparotomy was closed by a single running stitch (closing the abdominal muscle layer), using a resorbable 3–0 monofilament Vicryl suture (cutting blade needle). The wound was then closed with single loop sutures every 0.75 cm using the same suture. Sutures were checked before finally tying off and the surgical area was cleaned prior to recovery.

Recovery proceeded over a period of 30–50 min in an enclosed incubator at 30°C. Post-surgery, animals were returned to the pen once ambulatory. Rabbits were dosed with SC enrofloxacin (Baytril^®^; 5 mg/kg) antibiotic twice daily for 3 days post-surgery, and single doses of the analgesic buprenorphine (0.02 mg/kg) if displaying evidence of discomfort. Animals were typically active and feeding within 24 h.

To determine group size for the interventional studies, a power calculation was performed using the increase and variability of Masson’s trichrome staining data during model development (S1.1 Fig in S1 File). Eight rabbits underwent UUO; four were treated with intravenous (IV) rbBB7 100 mg/kg once every 5 days (i.e., one IgG half-life in rabbits) starting 1 day pre-UUO (Day 1), and thereafter on Days 4, 9, 14, and 19, with study completion on Day 25. The remaining four UUO rabbits received vehicle only and were compared against four sham operated rabbits for disease development. Rabbits were randomized to a dosing group sequentially post-surgery. Blood samples were taken immediately prior to each dose of antibody and at termination for pharmacokinetics. The animals were terminated by anesthetic overdose on Day 25, and the renal tissue harvested. Kidneys were halved longitudinally through the papilla; one half was fixed in 10% neutral buffered formalin for 24 h and paraffin embedded and the remaining half was cut transversely, with each segment immediately snap frozen in liquid nitrogen. All eight rabbits were included in the analyses; unless otherwise stated, all observations reported were conducted 25 days after surgery, post-termination.

#### Cynomolgus monkey

Eighteen previously untreated, male cynomolgus monkeys aged 7–8 years with no evidence of CKD (proteinuria, serum creatinine, urea) were randomized to UUO (n = 16) or sham operation (n = 2) groups. UUO was performed by Prisys Biotechnologies Co., Ltd, Shanghai, China, under study number IACUC-2015-PS11-002 (license code No.204 0000037). The UUO procedure has been described previously [34]. In brief, monkeys were confirmed free of tuberculosis and viral, bacterial, or parasitic infections (including simian immunodeficiency virus, respiratory syncytial virus, simian-T-lymphotropic virus, Schmallenberg virus, shigellosis, and salmonella) and selected based on urine albumin to creatinine ratio <2.5 mg/mmol, serum creatinine <125 μmol/L, and blood urea nitrogen <9 mmol/L at study initiation. The 18 monkeys recruited to the study were screened from 35 available animals to ensure they met the criteria defined above. Monkeys had free access to monkey chow (Guangzhou Guolong Science & Technology Co. Ltd., China) and municipal tap water meeting drinking standards. Vegetables and fruit treats were also given daily to stimulate their appetite. Each monkey was housed in a stainless-steel cage with a minimum living area of 160 x 80 x 190 cm (conforming to EU standards) at a temperature of 18–29°C and a relative humidity of 40–70%. Animals also had access to an exercise cage (480 x 80 x 190 cm) for 24 h each week. In addition, cages were equipped with enrichment devices including mirrors, balls, swings, ropes, and pictures that were regularly interchanged. Food treats were placed in devices that required the animals to work out how to access the treat.

Animals were fasted for 12 h, and water withheld for 3 h prior to general anesthesia. Anesthesia was induced by intramuscular (IM) injection of tiletamine and zolazepam (Zoletil^™^ 50 Virbac, Carros, France; 5 mg/kg) initially and maintained using 5% isoflurane inhalation. Prior to a midline incision in the abdominal wall, fur was shaved, and skin disinfected using an iodine-based scrub. The ureter of the left kidney was dissected and ligated at two adjacent points by silk ties (6–0 Prolene non-absorbable monofilament suture) and the abdominal wall subsequently closed. To minimize infection, penicillin (25,000 IU/kg) was given by IM injection twice daily for 3 days. Analgesia was provided with oral codeine (0.5 mg/kg) prior to surgery and then three times a day for 3 days post-surgery, and IM lornoxicam (0.7 mg/kg) was administered 20 min before surgery then 0.35 mg/kg twice daily for 3 days starting from 11 h post-surgery.

Post-surgery, the appearance, body weight, temperature, respiratory rate, and behavior of the animals were closely monitored and assessed daily. Skin wounds were evaluated by an onsite vet daily until the wound healed. UUO animal behavior was scored and recorded during each observation, and soft bedding was provided following surgery until their behavior returned to normal. In addition, an appropriately qualified and licensed representative from UCB Pharma was on site for all surgical procedures and for at least 7 days post-surgery. The representative ensured that high surgical standards were maintained and performed independent welfare checks on animals at least once a day, in addition to those performed by Prisys. All animals recovered from the UUO surgical procedure within a few days.

In total, 16 monkeys underwent the UUO procedure and two a sham operation (no statistical analyses were conducted using the two sham-operated animals). Monkeys undergoing UUO were sequentially randomly assigned to one of three treatment groups, receiving either formulation buffer (2 mL/kg, n = 6) or zampilimab at 10 mg/kg (n = 6), or 50 mg/kg (n = 4) IV every 7 days (IgG half-life in cynomolgus monkeys) starting on the day of surgery and ending on Day 21, with the study stopped on Day 28. Two non-operated monkeys received zampilimab 100 mg/kg with the same regimen to provide early safety data; in total 20 monkeys were included in this study. Experimental group size was determined by performing a power calculation using the increase and variability of Masson’s trichrome and Picrosirius red staining data during model development [34]. Plasma samples were taken at 0.25, 6, 24, 48, 96, 168, 168.25, 336, 336.25, 504, 504.25, 510, 528, 552, 600, and 672 h post-dosing for pharmacokinetics. At harvest, animals were euthanized by IV injection of pentobarbital (80 mg/kg); kidneys dissected longitudinally through the papilla, and each half cut into four transverse segments with each cut going through the papilla. One segment from each half kidney was fixed in 10% neutral formalin solution and embedded in paraffin blocks. The remaining segments were snap frozen in liquid nitrogen for later analysis. Unless otherwise stated, all observations reported were conducted 4 weeks after surgery, post-termination.

Toxicology and pathology methods are provided in the Part S2: Supplemental toxicology and pathology information in S1 File.

### Transglutaminase *in situ* activity assays

Detection of *in situ* TG activity was performed as previously described [35] with some modification. Frozen kidney tissue was mounted in optimal cutting temperature mounting media (VWR International, UK). Unfixed sections (10 μm) were washed in PBS and incubated with reconstitution PBS (5% BSA 5 μg/mL of streptavidin), with protease inhibitors for 30 min at RT. Sections were then washed twice with PBS prior to being incubated with reaction mix containing 5 mmol/L CaCl_2_, 1 mmol/L dithiothreitol, and a TG substrate (0.5 mmol/L biotin cadaverine [Thermo Fisher Scientific] or 1 μmol/L biotinylated T26 peptide [Zedira]) with protease inhibitors in 50 mmol/L Tris (pH 7.4) for 30 min at 37°C. A negative control in which CaCl_2_ was replaced with 10 mmol/L EDTA was used. Slides were washed three times with PBS, fixed in cold acetone for 5 min, and air dried. Sections were washed in PBS and incubated with a 1:500 dilution of streptavidin-Alexa Fluor 555 (Thermo Fisher Scientific) for 1 h at 37°C. Slides were then washed three times with PBS and mounted with anti-fade mounting medium containing DAPI.

### Circulating levels of anti-TG2 antibodies

In rabbit, plasma levels of rbBB7 were determined by enzyme-linked immunosorbent assay (ELISA) immunoreactivity to purified recombinant human TG2 (rhTG2). Ear vein bleeds were collected in EDTA tubes (Greiner Bio-One), and plasma prepared by centrifugation at 1000 x g for 15 min at 4°C. ELISA plates were coated with rhTG2 (50 ng/well; Zedira). Wells were washed in PBS/Tween (0.1% v/v), blocked with PBS/Tween 3% BSA (w/v) for 2 h at RT, washed, then plasma (diluted in PBS/Tween 1% BSA) was added and the plates incubated for 1 h at RT. Wells were washed and then incubated with anti-rabbit HRP 1:5000 in PBS/Tween 1% BSA for 1 h at RT and revealed with TMB substrate (Thermo Fisher Scientific). The reaction was stopped with 2 M H_2_SO_4_, and optical density determined by spectrophotometer at 450 nm. Negative control plasma from rabbits injected with vehicle alone did not exhibit detectable reactivity against rhTG2 in this assay.

In cynomolgus monkeys, zampilimab was measured in lithium-heparinized plasma samples by liquid chromatography–electrospray ionization–tandem mass spectrometry assay using zampilimab (isotopically labeled peptide H_2_N– GLPSSIEK–COOH) as internal standard for zampilimab. The method has a limit of quantification of 1 μg/mL for zampilimab.

### Protein staining and analysis

#### Hydroxyproline

In rabbits, this was performed as previously described [36]. In brief, a kidney homogenate from one kidney segment was generated, and 5 mg of protein (bicinchoninic acid assay) per sample were hydrolyzed in 6 mol/L HCl at 110°C for 24 h before neutralization with 12 M NaOH. Samples were centrifuged at 18,000 x g for 2 min and the supernatant transferred to clean tubes and freeze dried. Samples were then re-suspended in 500 μL of lithium loading buffer, and 30 μL was fractionated using a lithium chloride gradient on a Biochrom 30+ Amino Acid Analyzer (Biochrom, UK) using the manufacturer’s standard protocol; hydroxyproline content was expressed as μmol/mg protein.

In cynomolgus monkeys, two segments from each kidney were homogenized on ice in saline (0.9% NaCl) at 1:9 tissue:saline (w/v). Protein concentration in supernatant was determined using the Bradford assay. Kidney homogenate containing 10 mg of protein was mixed with concentrated HCl (38%, 12 M) at a ratio of 1:1 (v/v) to give a final HCl concentration of 6 M. Sample volume was topped up to 2 mL using 6 M HCl and samples incubated at 110°C for 18 h. After incubation, 1 mL 12 M NaOH solution was added to neutralize the acid, and the supernatant collected after centrifugation at 10,000 x g for 5 min. Hydroxyproline concentration in supernatants was measured by liquid chromatography–tandem mass spectrometry and was corrected by total protein and expressed as μM/mg protein. Briefly, hydroxyproline was measured with the Sciex API Triple Quad^TM^ 4500 System (Sciex, USA); sample separation by high-performance liquid chromatography was performed on an Eclipse XDB-C18 column (3.0 x 150 mm, 5 μm; Agilent, USA) with a mobile phase of water (with 0.1% formic acid) and acetonitrile (0.1% formic acid) at different flow rates; electrospray ionization was performed and ions were detected in multiple reaction monitoring mode (131.97/67.9 [declustering potential: 56, collision energy: 25]). Data were analyzed using the SCIEX OS analysis software Version 1.6.1.

#### Collagen I immunofluorescence

Formalin-fixed, paraffin-embedded rabbit and cynomolgus kidney sections were blocked with 5% BSA at 37°C for 30 min. Slides were incubated with primary antibody (goat anti-type-I collagen-UNLB; SouthernBiotech, USA; 1310–01) at a dilution of 1:1000 in 1% BSA/PBS, overnight at 4°C. Slides were then washed four times with PBS prior to incubating with donkey anti-goat IgG (H+L) cross-adsorbed secondary antibody conjugated to Alexa Fluor Plus 488 (Thermo Fisher Scientific; A32814), diluted 1:250 in 2 μg/mL DAPI in 1% BSA/PBS at 37°C for 1 h. Sections were washed four times in PBS prior to mounting in Mowiol^®^ mounting media.

#### Fibronectin immunofluorescence

Rabbit and cynomolgus kidney sections were blocked with 5% goat serum (Vector Laboratories, UK) and incubated with mouse anti-fibronectin (Millipore; MAB88916-C) diluted 1:400 in PBS overnight at 4°C. Slides were washed five times in PBS and incubated with goat anti-mouse–Alexa Fluor 568 (Thermo Fisher Scientific; A11031) diluted 1:250, for 1 h at RT. Slides were washed five times in PBS and mounted in DAPI-Mowiol^®^.

#### Cytochemical staining

Neutral-buffered, formalin-fixed, paraffin-embedded 4 μm sections were dewaxed and rehydrated. Nuclei were stained with Weigert’s hematoxylin, washed in water, and stained in Picrosirius red solution for 1 h prior to being washed in acidified water. Sections were dehydrated and cleaned using a series of alcohols and xylenes and then mounted in dibutylphthalate polystyrene xylene (DPX). Masson’s trichrome stain (which stains collagenous material blue, nuclei brown and fibers, erythrocytes, and elastin red/pink) was then performed using a staining kit (Sigma-Aldrich) as per manufacturer instructions.

#### Image acquisition and analysis

Whole-slide scans were acquired on either a NanoZoomer 2.0-HT Digital Slide Scanner (Hamamatsu, Japan) or a VS120-L100 Slide Scanner (Olympus, USA). For bright-field images (for Masson’s trichrome and Picrosirius red staining), scans were downloaded into Definiens Tissue Studio (Definiens Inc., USA). Selection of the region of interest for analysis (i.e., cortex only) was performed manually. Definiens Stain Picker options and ‘computer training/learning’ methods were used to generate an algorithm to identify differentially stained structures. The applied algorithms automatically calculated the area of the defined cortex and the defined differentially stained areas for each slide. The ratio of collagen area to cytoplasmic area was subsequently calculated to represent a fibrosis index. The dedicated Definiens Fluorescence workspace in Tissue Studio was used to analyze TG2 activity by immunofluorescent staining. Cortex detection was performed automatically and corrected manually to remove any incorrectly identified medulla from analysis. Marker detection was then performed on each image layer and the area of the staining for each fluorochrome determined. Levels of staining were then normalized by DAPI nuclear stain.

Manual pathological scoring of sections (using Masson’s trichrome-, Picrosirius red-, and hematoxylin- and eosin-stained sections as required) was performed by three experienced renal fibrosis scientists blinded to the experimental codes. A scoring system of 0 to 10 was employed, encompassing multiple parameters related to tubulointerstitial fibrosis only, due to the model used. Parameters included thickness of tubulointerstitial basement membrane, loss of brush border, flattening of proximal tubular epithelial cell, tubular architecture/dilation, tubular atrophy, number of infiltrating cells/proliferating fibroblasts, and overall tissue structure.

### Statistical analysis

Data are shown as mean ± standard deviation. Data analyses were performed using t-test, one- or two-way analysis of variance (ANOVA), followed by a Sidak or Tukey multiple comparisons test as appropriate, using GraphPad Prism Version 8.1 software. A probability of 95% (*p*<0.05) was regarded as significant.

## Results

### Humanization and characterization of TG2 inhibitory antibodies

Hybridoma clones mDC1 and mBB7 were selected as lead antibodies based on consistent reactivity to TG2, absence of reactivity to other key TG family member proteins (TG1, TG3, TG7, and Factor XIIIa), and ability to inhibit TG2 activity [23]. To reduce the risk of xenoreactive immune responses in future studies with patients, both antibodies were fully humanized (hDC1 and hBB7). Humanized antibodies had a half-maximal effective concentration (EC_50_) of ~20 pM (assessed by ELISA) for TG2 but did not bind to other TG family members (TG1, 3, 5, 7, and Factor XIIIa) up to concentrations of 100 nM. To facilitate *in vivo* studies, hBB7 was subsequently “rabbitized” as rbBB7.

To ensure humanization or rabbitization had not affected the antibodies’ functional characteristics, binding affinities for TG2 and the ability to block transamidation activity were assessed. The K_d_ for hDC1, hBB7, and rbBB7 from human, cynomolgus monkey, and rabbit TG2 were determined by surface plasmon resonance in the presence or absence of calcium. Both humanized antibodies showed high affinity (pM) for human and cynomolgus monkey TG2 irrespective of Ca^2+^ level, while rbBB7 also had good affinity against rabbit TG2 (Table 1). Calculation of IC_50_ values against recombinant protein in a KxD assay showed both hDC1 and hBB7 had potent inhibitory potential against both human and cynomolgus TG2 and were therefore more effective than the original mouse antibodies. hDC1 had fractionally higher inhibitory potential than hBB7 and was thus deemed the lead therapeutic candidate (zampilimab). rbBB7 had a 40-fold higher IC_50_ against rabbit TG2 than human, however, 8 nM is still considered a potent TG2 inhibitor (Table 2).

**Table 1:** Binding affinities of candidate antibodies for human, cynomolgus monkey, and rabbit TG2 by surface plasmon resonance.

	Geometric mean [range] K_d_, pM		
	Human TG2	Cynomolgus TG2	Rabbit TG2
**Zampilimab**^**a**^ **(–Ca**^**2+**^**)**	<107 [<102–112]	<50 [<32–62]	Biphasic
	n = 3	n = 2	n = 2
**Zampilimab**^**a**^ **(+Ca**^**2+**^**)**	<50 [<19–120]	<37 [<32–44]	Biphasic
	n = 5	n = 2	n = 2
**hBB7 (+Ca** ^**2+**^ **)**	<60 [<39–84]	<40	–
	n = 5	n = 1	
**rbBB7 (+Ca** ^**2+**^ **)**	<131	–	<185
	n = 1		n = 1

^a^Also referred to as hDC1.

n = number of replicates.

Ca^2+^, calcium ions; K_d_, dissociation constant; TG2, transglutaminase 2.

**Table 2:** Inhibition of human, cynomolgus monkey, and rabbit cell-surface TG2 activity in renal proximal tubule epithelial cell by the candidate antibodies.

	IC_50_ [95% CI], nM		
	Human TG2	Cynomolgus TG2	Rabbit TG2
**Zampilimab** ^**a**^	0.25 [0.20, 0.30]	0.22 [0.18, 0.27]	102.64 [62.86, 167.60]
**hBB7**	0.28 [0.23, 0.35]	0.25 [0.14, 0.43]	6.59 [5.54, 7.85]
**rbBB7**	0.30 [0.22, 0.40]	0.25 [0.19, 0.33]	8.36 [6.67, 10.48]

^a^Also referred to as hDC1.

Each value is the mean of five replicates. Activity measured using the biotin cadaverine incorporation assay.

CI, confidence interval; IC_50_, half-maximal inhibitory concentration; TG2, transglutaminase 2.

### Zampilimab inhibits extracellular TG2 activity and attenuates ECM accumulation in *in vitro* models of tubulointerstitial fibrosis

A primary human RPTEC model of tubulointerstitial fibrosis using TGF-β1 induction was established, and extracellular TG activity measured using a biotin cadaverine incorporation assay. From four independent studies, zampilimab had an IC_50_ of 119 nM.

**Fig 1:**
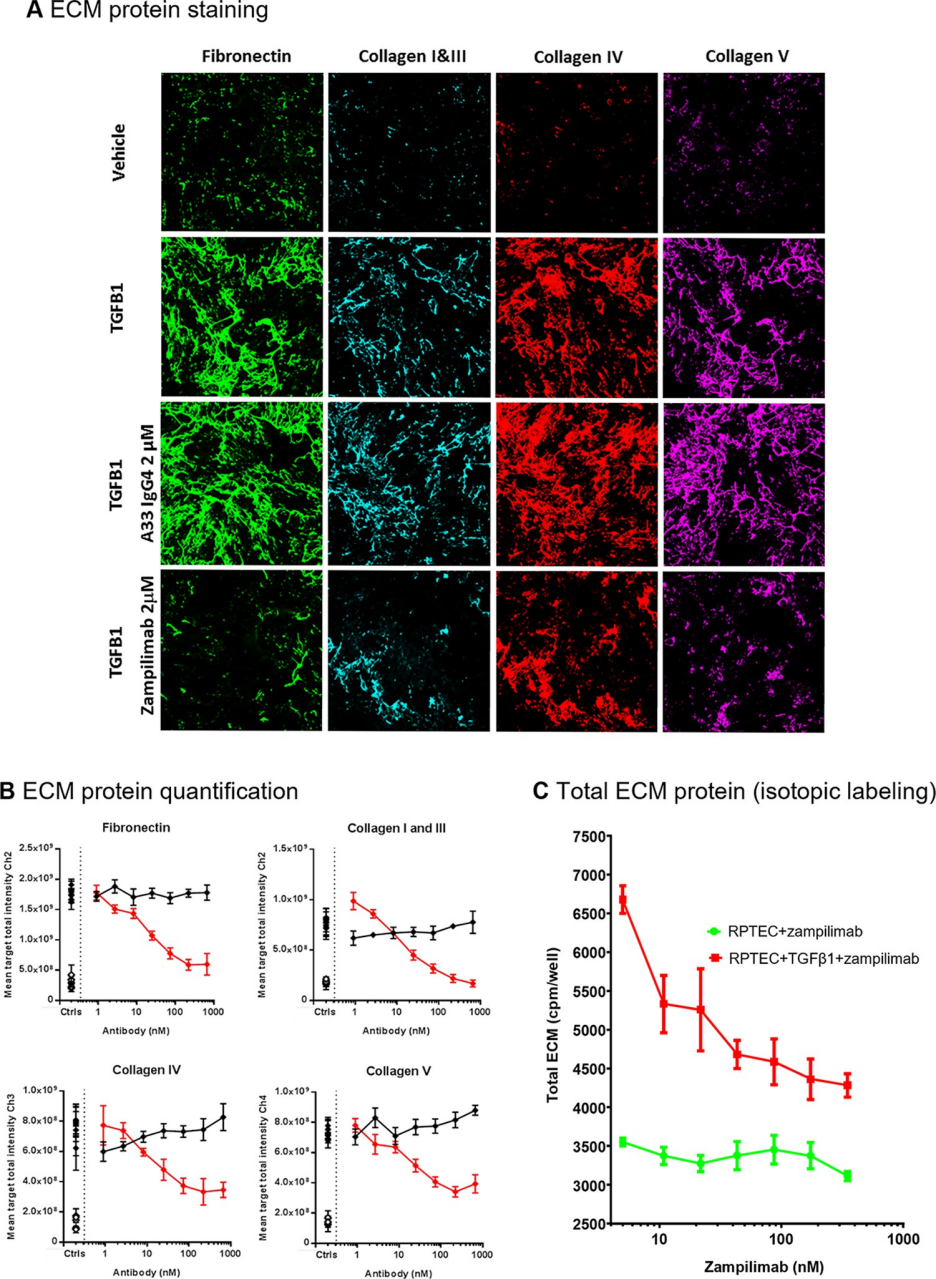
Zampilimab inhibits ECM accumulation in a primary human renal proximal tubule epithelial cell model of tubulointerstitial fibrosis. (A) Immunofluorescent staining of fibronectin (green), collagen I and III (blue), collagen IV (red), and collagen V (purple) in the ECM of RPTEC induced with TGF-β1 (30 ng/mL) and treated with either zampilimab 2 μM or isotype control antibody (A33 IgG4). Images are representative of a single experiment. (B) Quantification of fibronectin, collagen I and III, collagen IV, and collagen V staining in the ECM of RPTEC induced with TGF- β1 (30 ng/mL) with increasing concentrations of zampilimab (red) or isotype control antibody (A33 IgG4; black). The level of ECM proteins in unstimulated (open diamond -vehicle) and induced (closed diamond -TGF-β1) with no antibody added are in the Ctrls column. Results are shown from one of four replicate experiments with each plotted value calculated from eight technical repeats. Data = mean stain area ± SD. (C) Total ECM levels measured by tritiated amino acid incorporation in cell denuded ECM from both RPTEC and RPTEC induced with TGF-β1 that were subsequently treated with increasing concentrations of zampilimab. Data represent ECM levels based on ^3^H-incorporated amino acids (counts per well) ± SD (n = 4). Scale bar = 50 μm. Ch, channel; CPM, counts per minute; Crtl, control; ECM, extracellular matrix; ^3^H, tritium; Ig, immunoglobulin; RPTEC, renal proximal tubule epithelial cells; SD, standard deviation; TGF-β, transforming growth factor-β.

The same primary human cell model was used to analyze mature deposited ECM proteins. TGF-β1 induced the accumulation of collagens I, III, IV, V, and fibronectin by 3.5, 7, 8, and 4-fold respectively, compared with vehicle alone (Fig 1A and 1B). Zampilimab inhibited all ECM proteins with a mean IC_50_ ranging from 35 nM for collagen V through to 50 nM for fibronectin (Table 3, Fig 1A and 1B), and mean maximal response (E_max_) from 51% for collagen V to 134% for collagen I and III (Table 4; S1.2 Fig in S1 File). A control IgG4 antibody had no effect. The effect on total ECM levels was measured by incorporation of tritiated amino acids into the ECM. TGF-β1 increased the total amount of ECM produced by 88%. This amount was dose-dependently reduced by zampilimab, with an IC_50_ of ~30 nM. (Fig 1C; S1.3 Fig in S1 File). In cells that were not treated with TGF-β1, zampilimab had no effect on ECM build-up within the range of concentrations tested (0–90 nM).

**Table 3:** IC_50_ values for zampilimab on ECM accumulation in a primary human renal proximal tubule epithelial cell model of tubulointerstitial fibrosis stimulated with TGF-β1 (30 ng/mL) and in the presence of calcium (500 μM).

	IC_50,_ nM				
	*Run 1*	*Run 2*	*Run 3*	*Run 4*	Geometric mean
					[95% CI] IC_50_, nM
**Fibronectin**	58.94	59.68	44.67	41.03	50.39 [37.16, 68.33]
**Collagen I and III**	37.45	40.93	64.00	24.44	39.35 [21.00, 73.74]
**Collagen IV**	31.16	41.92	61.42	20.62	35.86 [17.20, 74.78]
**Collagen V**	43.64	35.86	36.96	26.23	35.10 [25.03, 49.21]

There were eight technical repeats for each condition within each experiment run.

CI, confidence interval; ECM, extracellular matrix; IC_50_, half maximal inhibitory concentration; TGF-β1, transforming growth factor-β1.

**Table 4:** E_max_ values for zampilimab on ECM accumulation in a primary human renal proximal tubule epithelial cell model of tubulointerstitial fibrosis stimulated with TGF-β1 (30 ng/mL) and in the presence of calcium (500 μM).

	E_max_, %				
	*Run 1*	*Run 2*	*Run 3*	*Run 4*	Mean [95% CI] E_max_, %
**Fibronectin**	87.95	110.70	93.94	187.80	120.1 [46.66, 193.5]
**Collagen I and III**	124.30	123.60	123.40	163.00	133.6 [102.4, 164.8]
**Collagen IV**	61.50	41.50	38.21	73.78	53.75 [26.92, 80.58]
**Collagen V**	46.12	35.86	53.36	68.48	50.96 [29.13, 72.78]

There were eight technical repeats for each condition within each experiment run.

CI, confidence interval; ECM, extracellular matrix; E_max_, maximal response; TGF-β1, transforming growth factor-β1.

**Fig 2:**
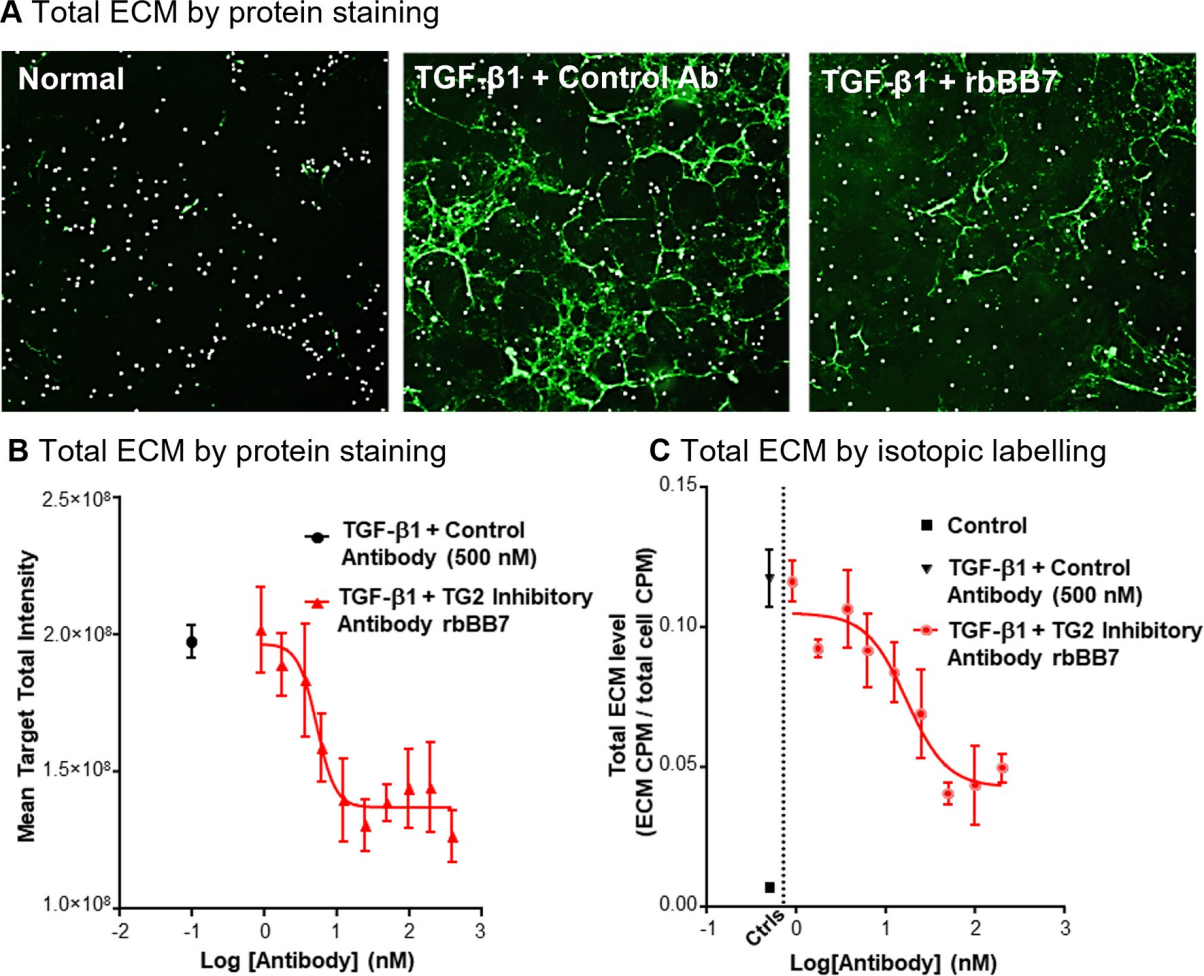
rbBB7 inhibits ECM accumulation in a rabbit primary co-culture model (RPTEC and fibroblasts) of tubulointerstitial fibrosis. (A) Fluorescent total ECM protein staining using flamingo pink in a rabbit RPTEC and fibroblast co-culture induced by TGF-β1 (10 ng/mL) and treated with either rbBB7 100 nM or control antibody. (B) Quantification of total fluorescent protein staining in a rabbit RPTEC and fibroblast co-culture induced by TGF-β1 (10 ng/mL) and treated with increasing concentrations of rbBB7. Data represent mean stain area (±SD; n = 4). (C) Quantification of total ECM by incorporation of radiolabeled amino acids into the ECM in a rabbit RPTEC and fibroblast co-culture induced by TGF-β1 (10 ng/mL) and treated with increasing concentrations of rbBB7. ECM levels at baseline (black square) and induced with TGF-β1, plus control antibody (inverted black triangle) are also shown. The CPM were used as an arbitrary measure of ECM level. Data represents mean (±SD; n = 4). Scale bar = 50 μm. Ab, antibody; CPM, counts per minute; Ctrls, controls; ECM, extracellular matrix; RPTEC, renal proximal tubule epithelial cells; rbBB7, rabbitized anti-TG2 tool antibody; SD, standard deviation; TG2, transglutaminase 2; TGF-β1, transforming growth factor-β1.

Similar experiments in a co-culture model of rabbit RPTECs and fibroblasts were undertaken using the rabbitized antibody rbBB7 to establish both a cross-species response and whether the same benefit was seen in the presence of primary renal fibroblasts. ECM accumulation was quantified using both radiolabeled amino acid uptake and flamingo pink staining. As in the human RPTEC monoculture assay, TGF-β1 induced ECM accumulation, which was inhibited dose-dependently in the presence of rbBB7 (Fig 2A; S1.4 Fig in S1 File). There was strong association between ECM measurements using the radiolabeling assay (Fig 2B) and total ECM protein staining (Fig 2C) assays.

### Anti-TG2 antibodies slow wound healing in scratch assay models

**Fig 3:**
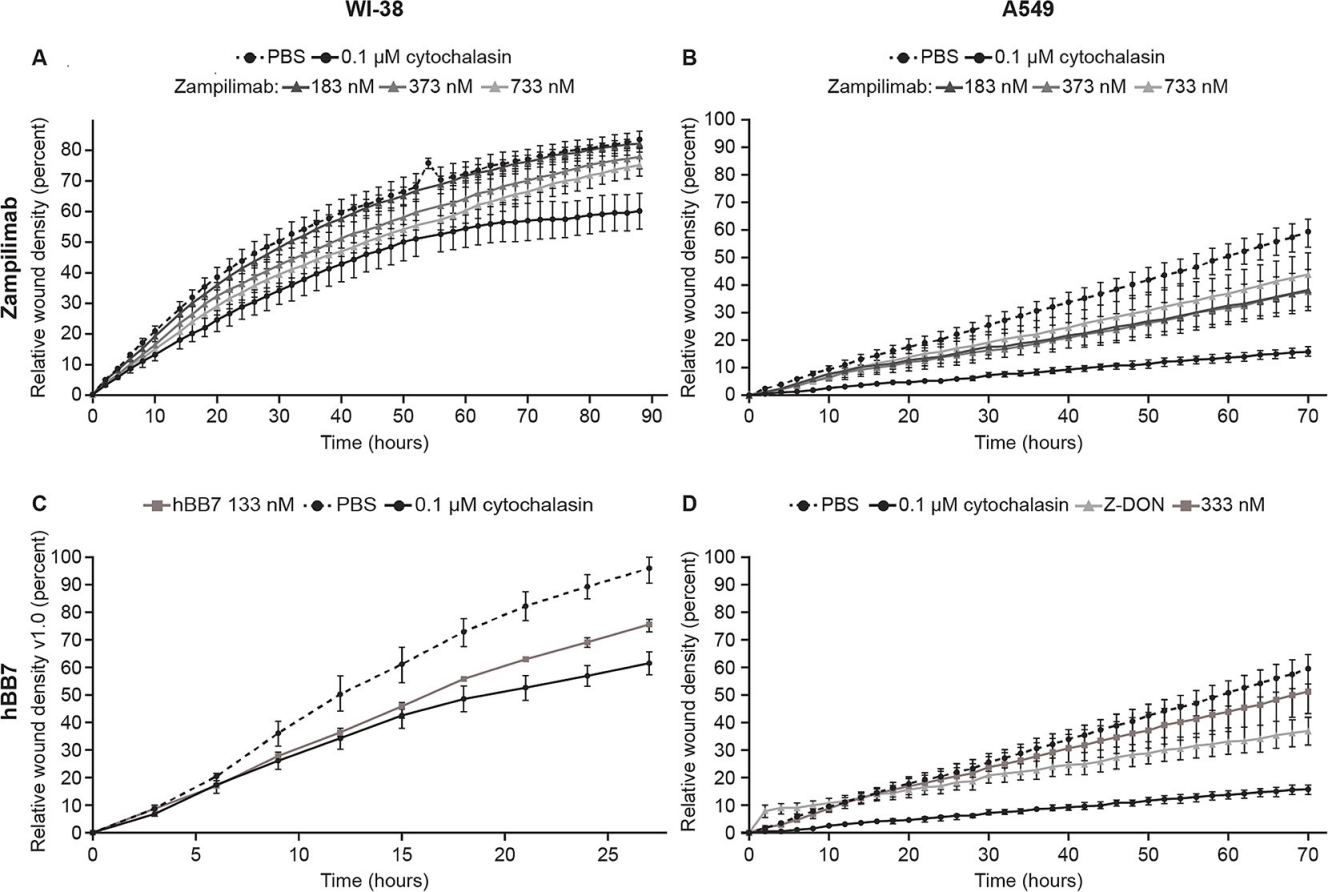
Scratch wound closure is delayed by TG2 inhibitory antibodies. Wound closure (assessed as mean wound density ±SD from >8 wells) with time in WI-38 (normal human lung fibroblasts) and A549 (adenocarcinomic human alveolar basal epithelial) cells treated with zampilimab, hBB7, cytochalasin D (0.1 μM, positive control), or PBS (negative control). Exemplar plots from >3 independent repeats. (A) WI-38 cells treated with zampilimab at 183, 373, and 733 nM. (B) A549 cells treated with zampilimab at 183, 373, and 733 nM. (C) WI-38 cells treated with hBB7 at 133 nM. (D) A549 cells treated with hBB7 at 333 nM using TG2 small-molecule inhibitor Z-DON as an additional positive control. PBS, phosphate-buffered saline; SD, standard deviation; TG2, transglutaminase 2.

In some tissues, such as lung, extracellular TG2 is predominantly catalytically inactive, but becomes transiently activated upon tissue injury as part of a wound-healing response [37], including aberrant wound healing or fibrosis. We assessed the effect of zampilimab and hBB7 on wound healing inhibition by quantifying the increase in wound density in normal lung fibroblasts and alveolar epithelial cell culture-based scratch assays (Fig 3). Both zampilimab and hBB7 slowed wound closure compared with a negative control in both cell types, with zampilimab demonstrating evidence of a dose response.

### rbBB7 reduces tissue fibrosis in a rabbit UUO model of CKD

Demonstrating that TG2 inhibitory antibodies could reduce ECM deposition and slow wound closure *in vitro* confirmed that extracellular inhibition of TG2 alone is sufficient to affect fibrotic potential. To confirm this *in vivo*, a UUO rabbit model of renal fibrosis was developed to facilitate treatment with rbBB7, as zampilimab does not sufficiently inhibit non-human TG2 (S1.1 Fig in S1 File). This rabbit model demonstrated progressive tubulointerstitial fibrosis (Masson’s trichrome and kidney hydroxyproline) over 28 days, that was also associated with increased TG2 activity from Day 7. In the subsequent interventional study, rbBB7 was administered at a high dose to compensate for reduced IC_50_ against rabbit TG2 (IV; 100 mg/kg). rbBB7 was injected 1 day before surgery, then every 5 days to Day 19 with study completion on Day 25. Four UUO rabbits received vehicle only; unless otherwise stated, all observations reported were conducted on Day 25.

### Pharmacokinetics of rbBB7

Trough plasma concentrations of rbBB7 increased after the first IV injection (approximately 500 μg/mL on Day 5; S1.5A Fig in S1 File). Trough plasma concentrations reached approximately 950 μg/mL by Day 15 (4^th^ dose). Pharmacokinetics were typical for an IgG antibody in rabbit.

### rbBB7 attenuated *in situ* TG2 Activity and reduced ECM deposition and renal fibrosis

Total TG activity, measured by cadaverine incorporation into cryo-sections, was elevated >10-fold by UUO. This increase was reduced by 70% in UUO animals receiving rbBB7. Using T26 peptide substrate (a preferential TG2 substrate) [38], there was a >20-fold increase in TG2 activity, lowered by 96% in the rbBB7 treatment group (S1.6 Fig in S1 File, *p*<0.00001, t-test), suggesting ~70% of TG activity in the UUO kidney is due to TG2, with ~30% due to other TG family members.

**Fig 4:**
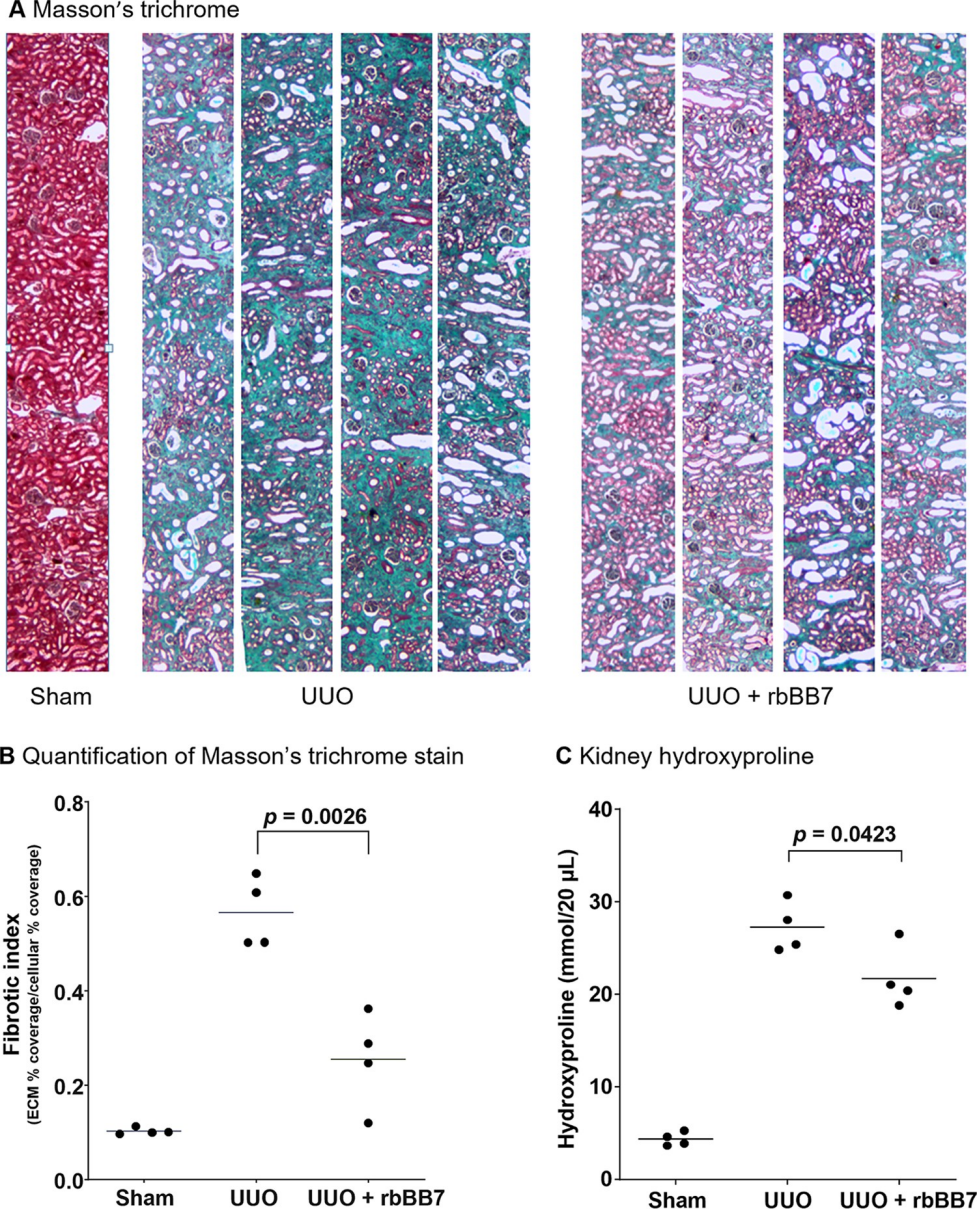
Renal fibrosis is reduced by rbBB7 in a rabbit UUO model of CKD. Eight New Zealand white rabbits were subjected to a left UUO and kept for 25 days for tubulointerstitial fibrosis to develop. Four UUO animals received no treatment (UUO) and four received 100 mg/kg of TG2 inhibitory antibody rbBB7 (UUO+rbBB7) every 5 days. Four additional animals received a sham operation (sham). (A) Masson’s trichrome staining showing a cortical slice at x40 magnification from each of the animals receiving UUO and an exemplar sham. Blue staining indicates collagen, red/pink indicates cellular areas. (B) Quantitative computerized image analysis of whole kidney scans of Masson’s trichrome staining of sham, UUO, and UUO + rbBB7 groups. Data shown are the mean fibrotic index, n = 4 per group with statistical significance calculated two-sample t-test. (C) Quantification of total kidney collagen by amino acid analysis of hydroxyproline from acid-hydrolyzed kidney samples from the sham, UUO, and UUO + rbBB7 groups. Each data point signifies a mean value from the analysis of two independent kidney segments from each animal. Data represent mean hydroxyproline concentration per 20 μL of a 10% renal homogenate, n = 4 per group with statistical significance calculated by two-sample t-test. Scale bar = 75 μm. CKD, chronic kidney disease; ECM, extracellular matrix; TG2, transglutaminase 2; UUO, unilateral ureteral obstruction.

The rabbit UUO model developed profound tubulointerstitial fibrosis consistent with hydronephrotic human kidneys [39]. By 25 days post-UUO, there was considerable expansion of the tubular basement membrane with significant epithelial flattening and tubular atrophy (Fig 4A). Administration of rbBB7 protected kidneys, with a visible reduction in collagen staining and provided protection from tubule loss, maintaining cortical architecture (Fig 4A). In a high-content image analysis of whole kidney scans, the 5-fold increase in fibrotic index due to UUO was reduced by 68% in the rbBB7-treated group (*p* = 0.026 Mann-Whitney U test; Fig 4B).

Tissue hydroxyproline concentrations were analyzed as a measure of overall collagen levels (Fig 4C). In untreated UUO rabbits, hydroxyproline increased approximately 7-fold at Day 25. Hydroxyproline in the rbBB7-treated group was 23% lower than in the untreated UUO kidney at Day 25 (*p* = 0.0423).

Collagen I, III, and fibronectin staining was assessed to further characterize the anti-fibrotic effect of rbBB7. All three ECM proteins were elevated with UUO; however, only collagen III (*p*<0.01) and fibronectin (*p*<0.0005, t-test) were significantly reduced by rbBB7 (S1.7 Fig in S1 File).

### Zampilimab reduced tissue fibrosis in a non-human primate UUO model of CKD

Positive rbBB7 data from the rabbit model justified the investigation of zampilimab effectiveness, given its IC_50_ is 40-fold lower against human TG2 than rbBB7 against rabbit. Zampilimab binds with high affinity and specificity to human and cynomolgus TG2 but shows little activity in other species. To identify any potential safety signals due to high-affinity TG2 blockade, studies with zampilimab in our non-human primate UUO model were necessary [34].

Cynomolgus monkeys undergoing UUO received formulation buffer (2 mL/kg, n = 6) or zampilimab (10 mg/kg [n = 6] or 50 mg/kg [n = 4]) IV every 7 days to Day 21, starting on the day of surgery, the study ended on Day 28. Two non-operated monkeys received zampilimab 100 mg/kg with same regimen to provide early safety data. Unless otherwise stated, all observations reported were conducted 4 weeks after surgery, post-termination.

#### Zampilimab reduced *in situ* TG2 activity and renal fibrosis in a primate UUO model

**Fig 5:**
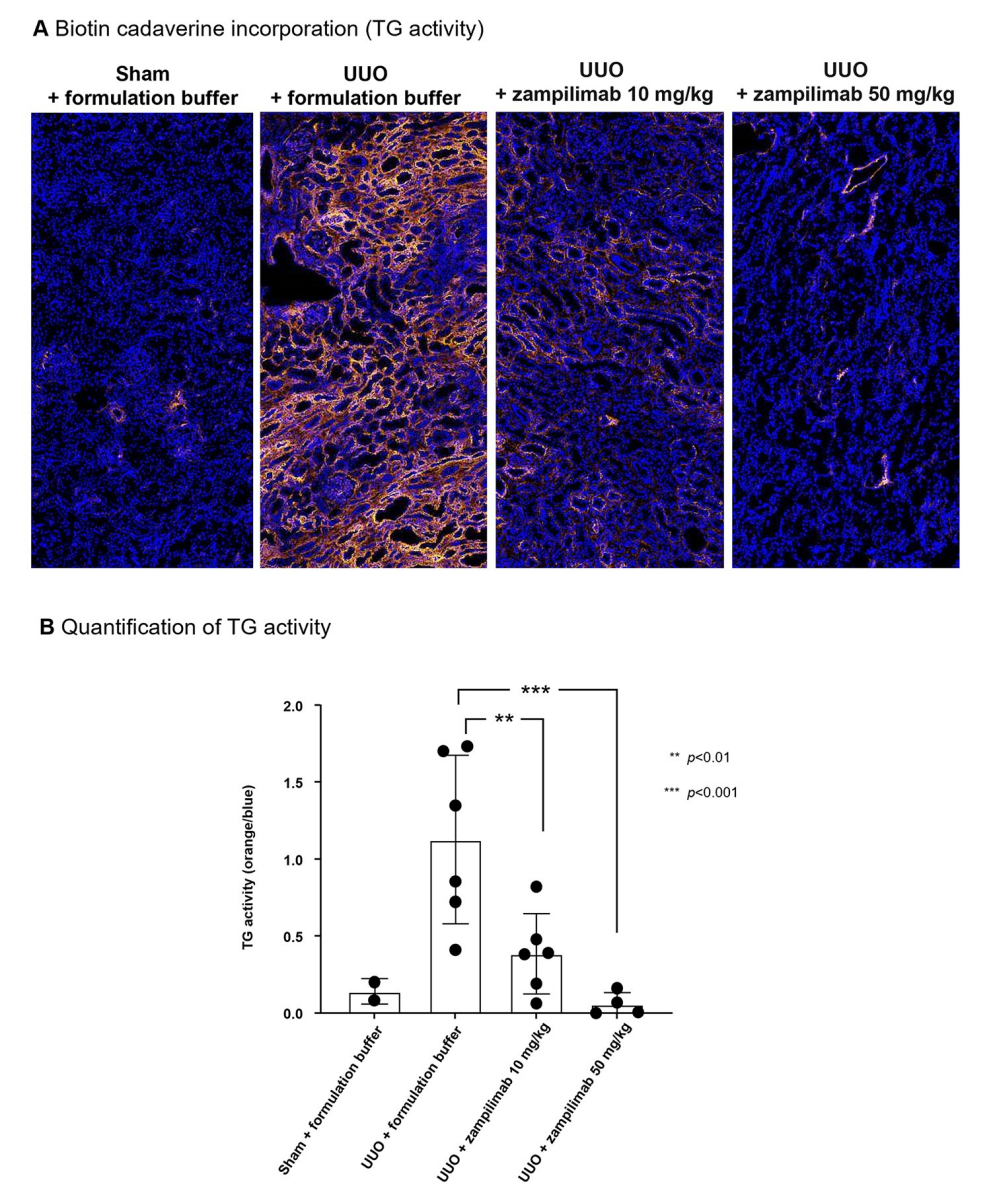
Zampilimab is an effective inhibitor of *in situ* renal TG2 activity in a cynomolgus monkey UUO model. Sixteen cynomolgus monkeys were subjected to a left UUO and kept for 28 days for tubulointerstitial fibrosis to develop. Six UUO animals received formulation buffer, six received 10 mg/kg of zampilimab, and four received 50 mg/kg of zampilimab every 7 days, starting on the day of surgery with the last dose on Day 21. Two additional animals received a sham operation (sham). (A) Visualization of TG2 activity by cadaverine incorporation in renal tissue sections from the sham, UUO, UUO + zampilimab 10 mg/kg, and UUO + zampilimab 50 mg/kg groups. Areas of positive cadaverine staining are shown as bright white. (B) Quantification of TG2 activity by cadaverine incorporation, calculated as the proportion of bright white versus gray staining. Statistical significance was performed using two-sample unequal variance (Welch) t-test to compare against the UUO control group. Scale bar = 50 μm. TG2, transglutaminase 2; UUO, unilateral ureteral obstruction.

TG activity (cadaverine incorporation) was approximately 10-fold higher in the kidneys of UUO monkeys than sham kidneys (Fig 5). UUO monkeys treated with zampilimab 10 mg/kg or 50 mg/kg had 70% (*p*<0.01) and 97% (*p*<0.001) reductions in TG activity, respectively, lowering TG2 activity to levels comparable with sham kidneys.

**Fig 6:**
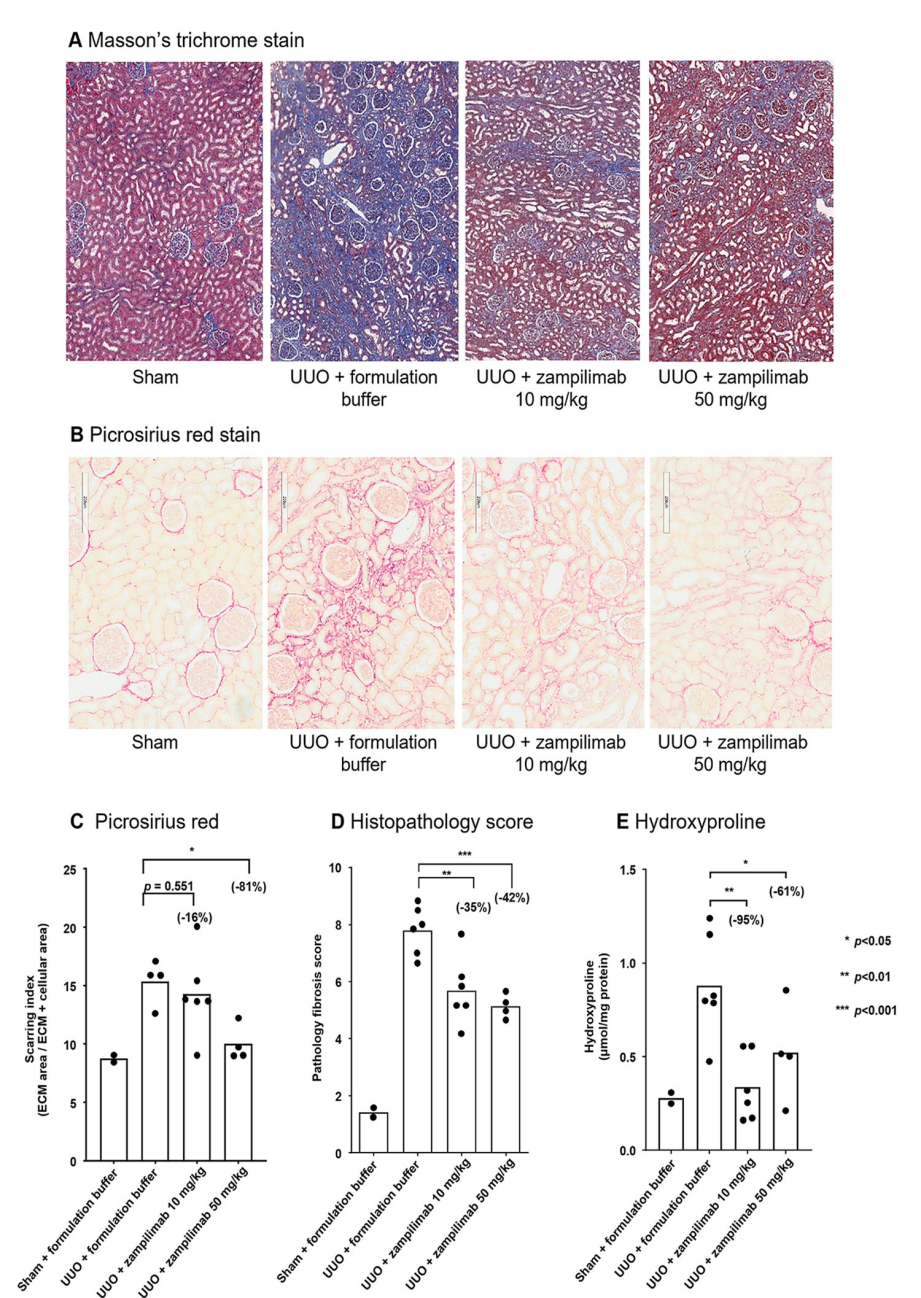
Effect of zampilimab on renal fibrosis in a cynomolgus monkey UUO model. Sixteen cynomolgus monkeys were subjected to a left UUO and kept for 28 days for tubulointerstitial fibrosis to develop. Six UUO animals received formulation buffer, six received 10 mg/kg of zampilimab, and four received 50 mg/kg of zampilimab every 7 days, starting on the day of surgery with the last dose on Day 21. Two additional animals received a sham operation (sham). (A) Masson’s trichrome staining showing a representative cortical image from each study group at x40 magnification. Blue staining indicates collagen, red/pink indicates cellular areas. (B) Picrosirius red staining showing a representative cortical image from each study group at x40 magnification. Red staining indicates interstitial collagen. (C) Quantitative computerized image analysis of whole section scans (approximately 1/8^th^ of kidney) of Picrosirius red staining of sham, UUO + formulation buffer, and UUO + zampilimab groups. Data shown are the mean scarring index, n = 2–6 per group with statistical significance calculated by one-way ANOVA (excluding sham–unequal variance). Two animals in the untreated UUO group and one in the zampilimab 10 mg/kg-treated group were excluded from analysis as they were not suitable for high-content imaging due to a fixation issue that affected the stain intensity. (D) Manual histopathological scoring of Masson’s trichrome stained renal tissue sections. Sections were scored by three blinded individuals using a 0–10 scale assessing pathological alterations in renal architecture. Mean score for each animal is plotted with bar representing mean value per group, n = 2–6 per group with statistical significance calculated by one-way ANOVA (excluding sham–unequal variance). (E) Quantification of total kidney collagen by mass spectrometry analysis of hydroxyproline from acid-hydrolyzed kidney samples from the sham, UUO + formulation buffer, and UUO + zampilimab groups. Each data point signifies a mean value from the analysis of two independent kidney segments from each animal. Data represent mean hydroxyproline concentration (μmol/mg protein), n = 2–6 per group with statistical significance calculated by one-way ANOVA (excluding sham–unequal variance). Scale bar = 75 μm on Masson’s Trichrome images (A) and 200 μm on Picrosirius red images (B). ANOVA, analysis of variance; ECM, extracellular matrix; UUO, unilateral ureteral obstruction.

In all UUO monkeys, kidney tubule sections showed notable tubular dilation, tubular atrophy, tubular epithelial flattening, brush border loss, and tubulointerstitial expansion. However, visual comparison of sections from different UUO groups suggested that the tubulointerstitial fibrosis was much less progressed in the zampilimab groups compared with the UUO + buffer group using both Masson’s trichrome (Fig 6A) and Picrosirius red (Fig 6B) staining. This difference was greatest in the UUO + 50 mg/kg group, suggesting that zampilimab dose-dependently preserved renal tubule structure.

Fibrosis assessed by high-content image analysis of Masson’s trichrome-stained sections showed the UUO + zampilimab 10 mg/kg group had a fibrosis index reduced by 16% (*p* = 0.551), while in UUO + 50 mg/kg groups an 81% (*p*<0.05) reduction was seen (Fig 6C). These data were confirmed by manual scoring of fibrosis in the tissue sections by three-blinded individual experts in the field, showing 35% (*p*<0.01) and 42% (*p*<0.001) reductions in the pathology score with zampilimab 10 and 50 mg/kg, respectively (Fig 6D).

Kidney tissues were further analyzed for hydroxyproline content by mass spectrometry, with lower hydroxyproline content in both zampilimab groups (10 mg/kg: 95% reduction, *p*<0.01; 50 mg/kg: 61% reduction, *p*<0.05) compared with the vehicle-treated UUO animals (Fig 6E).

To assess wound healing effects, dermal punch (3 mm) and linear wounds (10 mm) were introduced into treated cynomolgus monkeys (both UUO [50 mg/kg] and sham [100 mg/kg]) 1–28 days prior to the end of the study. Skin was recovered at termination and was microscopically examined for delayed wound closure. There was no obvious microscopic difference in the closure or cell infiltration into the wounded area at any of the examined time points (six wounds per animal; S1.8 Fig in S1 File). Additionally, in a subsequent phase I enabling 13-week toxicity study, a similar assessment of dermal wound closure was undertaken, which confirmed zampilimab had no effect on dermal wound healing.

### Pharmacokinetics of zampilimab

Zampilimab plasma concentrations increased in a dose-dependent manner and peaked at 6 h post-dose in cynomolgus monkeys (S1.5B Fig in S1 File). Zampilimab accumulation was noted after each administration, except in one animal in the UUO + 50 mg/kg group, in which concentrations of zampilimab dropped from 412 μg/mL 96 h after Dose 1, to 18.5 μg/mL 96 h after Dose 4; the reason for this is unclear, although anti-drug antibodies were not detected.

### No potential safety signals were observed with zampilimab

No clinical signs attributable to zampilimab were observed during the treatment phase. Similarly, no changes in organ histology, hematology, clinical chemistry, or urinalysis parameters were associated with zampilimab administration in UUO or control monkeys treated with zampilimab 100 mg/kg (S2.6–S2.45 Tables in S2 File), which were histologically normal (S1.9 Fig in S1 File).

## Discussion

In this study, the most potent murine TG2 inhibitory antibodies previously identified [23] were successfully humanized, lowering the IC_50_ and K_d_ compared with the original murine antibodies. The most successful of these antibodies, (hDC1/UCB7858/zampilimab) was highly effective in a human primary cell model of renal tubulointerstitial fibrosis, confirming that targeting only extracellular TG2 is sufficient to modify ECM accumulation. In scratch wound assays, both zampilimab and hBB7 recapitulated the slow wound closure seen with cells from TG2 KO animals [40] and the use of small molecule inhibitors [16], further supporting the extracellular crosslinking role of TG2 in wound healing.

Rabbitized BB7, despite having a higher IC_50_ against rabbit TG2 than zampilimab (against human TG2), was equally effective at blocking ECM accumulation in a primary rabbit co-culture cell model of tubulointerstitial fibrosis. Application of this antibody *in vivo* demonstrated the expected pharmacokinetics (i.e., with no target-mediated clearance and no apparent development of anti-drug antibodies over the period evaluated) in a rabbit UUO model with a profound antifibrotic effect, lowering the accumulation of ECM and strongly preserving tubular architecture. The success of rbBB7 in rabbit provided ethical justification to apply the most effective and primate-specific antibody, zampilimab, in a previously developed cynomolgus monkey UUO CKD model [34]. Zampilimab was effective at reducing tubulointerstitial fibrosis at both doses assessed, although the higher 50 mg/kg dose showed stronger TG2 activity inhibition and superior, less variable, protection against fibrosis compared with the 10 mg/kg dose. Pharmacokinetics were as expected for an IgG4P antibody in a cynomolgus monkey, with no evidence of anti-drug antibodies (either from plasma levels or emergence of anti-drug antibodies), nor safety signals observed even in normal cynomolgus monkeys that were treated in parallel with zampilimab 100 mg/kg.

It was important to demonstrate efficacy of zampilimab and BB7 on fibrosis to confirm mechanism of action and to verify that the inhibition of extracellular TG2 was sufficient to mediate anti-fibrotic activity. Several tool TG2 small-molecule inhibitors have been tested *in vitro* and *in vivo* with variable inhibition of other TG family members (including TG1, 3, 5, 6, and Factor XIIIa) [16, 35, 36, 41]. These molecules have been shown to have beneficial effects including prevention of kidney function decline [36], decreased renal scarring [35], reduced collagen deposition [41], and slower wound healing [16]. TG2 small molecule inhibitors act on both intra- and extra-cellular TG2, producing an environment similar to that in TG2 KO animals. TG2 has intracellular roles linked to guanosine triphosphatase (GTPase) activity [42], in particular the binding of nuclear factor kappa B (NF-κB) binding protein and facilitation of NFκB signaling, which appear crucial in oncology [43]. Previously this has cast doubt as to whether only blocking TG2’s extracellular crosslinking role would prevent fibrosis, especially as rapid inactivation of externalized TG2 has been reported in some tissues [37]. From these data, it is clear that preventing fibrosis by inhibiting extracellular TG2 (at least in the kidney) using targeted antibodies in a UUO model, is equivalent or better than in UUO models in TG2 KO animals. TG2-specific inhibitory antibodies therefore provide significant benefits over small-molecule inhibitors in terms of specificity issues due to the high catalytic core conservation across the eight active family members, which small molecule inhibitors struggle to differentiate between [36, 41].

Zampilimab and BB7 neither bind nor inhibit other TG family members, since the binding epitope is unique to TG2 [23]. Specific TG2 small molecules do exist, but are currently compromised by bioavailability, such as those described by the Huntington foundation [44], which do not have pharmacokinetic properties suitable for *in vivo* application. Zedira designed ZED1227 (phase IIb, Celiac disease) for topical application, but have gone on to develop a new class of ketoamide-based inhibitors (Patent WO2018122419A) that seek to improve *in vivo* oral bioavailability and pharmacokinetic properties beyond those of ZED1227 to allow systemic application. However, little is known about these molecules at this stage, especially how they perform in *in vivo* models of disease [45].

If TG2 inhibitors (small molecule or antibody) are not specific, potential off-target TG inhibition effects can occur, such as parakeratosis from inhibition of TG1, 3, and 5 [46], bleeding disorders following inhibition of Factor XIIIa [6], and neuron disorders relating to TG6. However, the fact that antibodies do not enter the cell but confer the same protection is a critical advantage, with no potential for toxicity from affecting TG2’s intracellular roles in NFκB signaling [43], GTP signaling [42], nuclear membrane function, and cytoskeleton crosslinking. The exquisite selectivity of these antibodies, with no binding to other TG family members, coupled with binding to extracellular proteins only, limits the risk of adverse events [47].

The strong human specificity of these antibodies with some reactivity against rabbit had severely restricted utility of the *in vivo* models available, leading us to develop renal models in rabbit and non-human primates for the first time. The UUO model in the current study is one of the simpler ways to transfer between species and is to some degree a relevant animal model of human CKD, replicating many features of obstructive nephropathy; however, it is not a functional model. Although development of a functional model would have been preferable, this could not be ethically justified in non-human primates due to the number of animals required, the variability, and the duration of functionality of the models. Attempts were made to develop such models in rabbits: a nephrotoxic serum nephritis model was developed but proved too variable for assessment of therapeutics. An aristolochic acid model was also developed with some success; however, the slow development of the disease meant it could not be applied beyond biomarker analysis. That said, TG small-molecule inhibitors have been used successfully in 5/6^th^ subtotal nephrectomy and streptozotocin-induced diabetic kidney disease models, with histologically similar protection of around 50% to that seen in the UUO models here, suggesting robust functional protection would also be seen. Importantly, we can extrapolate data from non-human primates to guide the dosage needed for humans. Inhibiting enzyme activity with an antibody can be challenging, often requiring uneconomical doses. The exceptionally low IC_50_, picomolar K_d_, quick on-rates, and remarkably long off-rates of zampilimab theoretically address this, but access to the ECM space, especially in a fibrotic tissue, is an unknown. These data clearly demonstrate that even at a 10 mg/kg dose (equivalent to 750 mg/kg every 4–6 weeks in humans), zampilimab has a clinically meaningful effect.

In the rabbit studies, comparisons of pan- and TG2-preferred substrates show that around 70% of the total extracellular renal TG activity is due to TG2. In the cynomolgus monkey, activity due to TG2 appears to be around 97%, which seems high compared with studies that observed TG2 in the human kidney (activity around 80%). This raises the question of whether TG activity from other TG family members can drive pathology; however, only two TGs (TG2 and Factor XIIIa) are known to be extracellular. The TG2 KO mouse was highly protected against the development of fibrotic lesions in a model of CKD by Shweke et al [48], and studies in a rodent model using a pan TG inhibitor (NTU283) demonstrated levels of protection comparable to the KO model [36]. Together, these studies suggest that total and TG2-specific knockdown provide comparable levels of protection from fibrosis, and hence TG2 is the key enzyme involved in pathology. The rabbit and non-human primate studies presented here showed consistent levels of protection against fibrosis, and in combination with the TG2 KO mouse data, further support the hypothesis that TG2 is the key driver of pathology. Although studies show that Factor XIIIa can crosslink fibronectin and collagen [49], specific activation of Factor XIII would be required, which normally only occurs under clotting conditions. Furthermore, there is no evidence in the literature of Factor XIIIa being pro-fibrotic, but rather there is some evidence of Factor XIIIa deficiency driving cardiac fibrosis [50]. It therefore appears that TG2 is the only TG family member involved in fibrotic remodeling.

Given its mode of action, inhibition of TG2 is expected to interfere with wound healing. Anti-fibrotic treatments pirfenidone and nintedanib, approved therapies for lung fibrosis, have demonstrated minimal effect on normal wound repair; however, benefit-risk must be assessed for every approach [51, 52]. The scratch wound closure assays used in this study suggest a TG2 inhibitor may carry a risk to normal wound healing, as suggested by prior studies using various fibroblasts from TG2 KO mice and TG2 siRNA studies [19]. Additionally, evidence from a study using topical application of putrescine (a non-specific TG inhibitor) on rat dermal wounds suggests that TG2 loss affects wound tensile strength [53]. To understand this risk with zampilimab, we carried out dermal punch and linear wound investigations in treated cynomolgus monkeys and microscopically examined the wounds over time. There was no obvious microscopic difference in the closure of, or cell infiltration into, the wounded area, although the tensile strength was not measured. Additionally, in the 13-week toxicity study, no clear differences in wound healing between control and zampilimab-treated animals were reported.

Inhibition of TG2 will likely not reverse existing fibrotic lesions, due to its mechanism of action. TG2 inhibition may protect healthy tissue within the organ from undergoing fibrotic modeling, thereby preventing progression of disease, which the prophylactic application of zampilimab employed in this study aimed to demonstrate. Although the data showed a clear prevention of fibrosis with zampilimab, the experimental design of the study did not address the effect of late therapeutic dosing; therefore, the ability of late zampilimab application to halt or reverse well-established disease has not been shown. Additionally, therapeutic dosing in a UUO model is challenging due to hydronephrosis and interstitial tissue loss, coupled with late ECM accumulation. Similarly, the *in vitro* models also have late ECM accumulation, accelerating confluency and impacting the interpretation of late-dosing experiments. Thus, the authors believe that treatment in established disease is best evaluated in clinical studies.

In conclusion, zampilimab inhibited extracellular TG2 activity both *in vitro* and *in vivo*, reducing accumulation of ECM components, slowing wound healing in human cells, and reducing fibrosis development in a cynomolgus monkey UUO model of CKD. Together, these findings support the further development of zampilimab for the treatment of human fibrotic disease. A phase I study (UP0105; NCT04705350) assessing safety, tolerability, and pharmacokinetics of zampilimab in healthy volunteers was recently completed successfully and is supportive of further clinical development in fibrotic diseases.

## Supporting information

S1 File Supplemental tables (S1.1–S1.4 Tables) and figures (S1.1–S1.9 Figs), and supplemental toxicology and pathology information (S2.1–S2.5 Tables; S2.1 Fig). (PDF)

S2 File Additional supplemental toxicology and pathology tables (S2.6–S2.45 Tables). (XLSX)
