# Supplementary material for: The effect of TG2-inhibitory monoclonal antibody zampilimab on tissue fibrosis in human in vitro and primate in vivo models of chronic kidney disease
Source: PLoS One. 2024 May 16;19(5):e0298864. doi: 10.1371/journal.pone.0298864 (PMC11098434; doi:10.1371/journal.pone.0298864)
Supplement: S1 File — (PDF) [file pone.0298864.s001.pdf]

<u>Supporting information</u>

The effect of TG2-inhibitory monoclonal antibody zampilimab on tissue fibrosis in human *in vitro* and primate *in vivo* models of chronic kidney disease

Linghong Huang, Helene Bon, Mabrouka Maamra, Toby Holmes, John Atkinson, Katharine Cain, Jeff Kennedy, Catherine Kettleborough, David Matthews, Breda Twomey, Jia Ni, Zhizhan Song, Philip F. Watson, Timothy S. Johnson

Contents

Part S1: Supplemental Tables and Figures 4

S1.1 Table. Heavy chain variable region framework sequences for murine BB7, DC1, and acceptor human framework AF062260. 4

S1.2 Table. Kappa light chain variable region framework sequences for murine BB7, DC1, and acceptor human framework A193851. 5

S1.3 Table. Sequence alignments of the final humanized heavy-chain versions. 6

S1.4 Table. Sequence alignments of the final humanized versions of kappa light chains. 7

S1.1 Fig. Development of a UUO model in the New Zealand white rabbit......8

S1.2 Fig. ECM protein accumulation is inhibited by zampilimab in a primary human renal proximal tubule epithelial cell model of tubulointerstitial fibrosis: percentage inhibition................................10

S1.3 Fig. TG2 inhibitory antibody zampilimab inhibits total ECM accumulation in a primary human renal proximal tubule epithelial cell model of tubulointerstitial fibrosis: percentage inhibition.........11

S1.4 Fig. TG2 inhibitory antibody rbBB7 inhibits total ECM accumulation in a primary rabbit renal proximal tubule epithelial cell and renal fibroblast co-culture model of tubulointerstitial fibrosis: percentage inhibition. 12

S1.5 Fig. Trough plasma concentrations of (A) rbBB7 in a rabbit UUO model and (B) zampilimab in a cynomolgus monkey UUO model show expected pharmacokinetics...13

S1.6 Fig. rbBB7 is a potent inhibitor of in situ TG2 activity in a rabbit UUO model of CKD………………………………………………………15

S.17 Fig. rbBB7 prevents accumulation of collagen III and fibronectin in a rabbit UUO model of CKD…………………………………………………17

S.18. Fig. Effect of zampilimab on dermal wound closure in cynomolgus monkeys…………………………………………………………….…19

S1.9 Fig. Zampilimab does not affect normal kidney histology in cynomolgus monkeys...21

Part S2: Supplemental toxicology and pathology information 22

Methods: Toxicology 22

Hematology and clinical chemistry...22

Hematology……………………………………………………….....22

S2.1 Table. Hematology parameters measured. 22

Clinical chemistry on serum..23

S2.2 Methods Table. Clinical chemistry parameters measured in serum......23

Urinalysis...24

S2.3 Methods table. Urine analysis parameters...24

Methods: Pathology...25

Necropsy and tissue preservation 25

Histology/pathology...25

S2.4 Methods Table. Tissue list. 26

Results: Toxicology and pathology 28

Raw toxicology and pathology data..28

Microscopic examination of tissue samples 28

Clinical pathology..29

S2.1 Fig. Effect of UUO and weekly zampilimab treatment for 4 weeks on serum levels of urea, creatinine, calcium, and phosphate. 32

Bone marrow and blood smear examination......................................33

S2.5 Table. Animal list for bone marrow and blood smear analysis. 33

Conclusion..34

Note: S2.2.6 – S2.45 Tables are located in the additional Excel file

Part S1: Supplemental Tables and Figures

S1.1 Table. Heavy chain variable region framework sequences for murine BB7, DC1, and acceptor human framework AF062260.

```
Kabat Numbers[2]        1       10        20        30          40        50           60        70        80           90       100                    110
                      -|--------|---------|---------|-----AB----|---------|--ABC-------|---------|---------|--ABC-------|---------|ABCDEFGHIJK---------|-
Vernier[4]            -.*........................****..................***....................*.*.*.*....*.................**.................*..........
Canonical[5]          -.......................1.11.1....1....................2...22...............2.........................1............................
Interface[6]          -..................................I...I.I.....I.I.................................................I.I.I................I..........
5Å Proximity           ****                 * *  ****       ***        ***                   ****** *    *               ****                 **
CDR                                                  <----->              <----------------->                                <---------------->
BB7_VHc (mBB7001VH)   -AVQLVESGGGLVKPGGSLKLSCAASGIIFSSSAMS--WVRQTPEKRLEWVATISS--GGRSTYYPDSVKGRFTVSRDSAKNTLYLQMDSLRSEDTAIYYCAKLI------------SPYWGQGTTLTVSS
DC1_VHc (mDC001VH)    -EVQLVESGGGLVKPGGSLKLSCAASGFTLSTHAMS--WVRQTPEKRLEWVATISS--GGRSTYYPDSVKGRFTISRDNVKNTLYLQLSSLRSEDTAVYFCARLI------------STYWGQGTTLTVSS
AF062260              C....L.......Q.....R.........F..Y...--....A.GKG....SA..G--S..S...A.............S...........A....V.....DG------------GV......LV....
```

Showing the alignment and residue identity of AF062260 to the murine antibodies. Residue identities are shown by a dot (.) character. Residue differences are shown where applicable. Gaps (-) are used to maintain Kabat numbering, and to show residue insertion or deletion where applicable. The “5Å Proximity” residues are indicated by *.

S1.2 Table. Kappa light chain variable region framework sequences for murine BB7, DC1, and acceptor human framework A193851.

```
Kabat Numbers2         1       10        20              30        40        50        60        70        80        90             100
                      -|--------|---------|-------ABCDEF--|---------|---------|---------|---------|---------|---------|-----ABCDEF----|------A-
Vernier4              -.*.*....................................**.........****..............*.*.**.*................................*..........
Canonical5            -.1......................1.......1111..1..............2..22...........2......1..................3....3...................
Interface6            -.........................................I.I.....I..........................................I..I............II..........
5Å Proximity           ******               **                 **         ****       ***  ***********              **               **
CDR                                         <--------------->               <----->                                <------------->
BB7_VKc (mBB001VK)    -AIKMTQSPSSMYASLGERVIITCKASQ------DINSYLTWFQQKPGKSPKTLIYLTNRLMDGVPSRFSGSGSGQEFLLTISGLEHEDMGIYYCLQYVDFP------YTFGGGTKLEI-K
DC1_VKc (mDC001VK)    -DITMTQSPSSIYASLGERVTITCKASQ------DINSYLTWFQQKPGKSPKILIYLVNRLVDGVPSRFSGSGSGQDYALTISSLEYEDMGIYYCLQYDDFP------YTFGGGTKLEI-K
AF193851              -...M......LS..V.D......R...------G.RN..A........A..S...AASN.QS............T..T......QP..FAT...Q.HNTY.------W...Q...V..-.
```

Showing the alignment and residue identity of AF193851 to the murine antibodies. Residue identities are shown by a dot (.) character. Residue differences are shown where applicable. Gaps (-) are used to maintain Kabat numbering, and to show residue insertion or deletion where applicable. The “5Å Proximity” residues are indicated by *.

S1.3 Table. Sequence alignments of the final humanized heavy-chain versions.

```
Kabat Numbers           1       10        20        30          40        50           60        70        80           90       100                    110
                       -|--------|---------|---------|-----AB----|---------|--ABC-------|---------|---------|--ABC-------|---------|ABCDEFGHIJK---------|-
Vernier[4]             -.*........................****..................***....................*.*.*.*....*.................**.................*..........
Canonical[5]           -.......................1.11.1....1....................2...22...............2.........................1............................
Interface[6]           -..................................I...I.I.....I.I.................................................I.I.I................I..........
5Å Proximity            ****                 * *  ****       ***        ***                   ****** *    *               ****                 **
CDR                                                   <----->              <----------------->                                <--------------->
BB7_RHB (hBB001HB)     -EVQLLESGGGLVQPGGSLRLSCAASGIIFSSSAMS--WVRQAPGKGLEWVATISS--GGRSTYYPDSVKGRFTVSRDSSKNTLYLQMNSLRAEDTAVYYCAKLI------------SPYWGQGTLVTVSS
DC1_RHB (hDC001HB)     -EVQLLESGGGLVQPGGSLRLSCAASGFTLSTHAMS--WVRQAPGKGLEWVATISS--GGRSTYYPDSVKGRFTISRDNSKNTLYLQMNSLRAEDTAVYFCARLI------------STYWGQGTLVTVSS
```

Showing the sequence alignments of the final humanized versions of BB7 and DC1 heavy chains. Gaps (-) are used to maintain Kabat numbering, and to show residue insertion or deletion where applicable.

S1.4 Table. Sequence alignments of the final humanized versions of kappa light chains.

```
Kabat Numbers          1       10        20              30        40        50        60        70        80        90             100
                      -|--------|---------|-------ABCDEF--|---------|---------|---------|---------|---------|---------|---------|-----ABCDEF----|------A-
Vernier[4]            -.*.*....................................**.........****..............*.*.**.*................................*..........
Canonical[5]          -.1......................1.......1111..1..............2..22...........2......1..................3....3...................
Interface[6]          -.........................................I.I.....I..........................................I..I............II..........
5Å Proximity           ******               **                 **         ****       ***  ***********              **               **
CDR                                         <--------------->               <----->                                <------------->
BB7_RKB (hBB001KB)    -DIKMTQSPSSLSASVGDRVTITCKASQ------DINSYLTWFQQKPGKAPKTLIYLTNRLMDGVPSRFSGSGSGQEFLLTISSLQPEDFATYYCLQYVDFP------YTFGQGTKVEI-K
DC1_RKB (hDC001KB)    -DITMTQSPSSLSASVGDRVTITCKASQ------DINSYLTWFQQKPGKAPKILIYLVNRLVDGVPSRFSGSGSGQDYALTISSLQPEDFATYYCLQYDDFP------YTFGQGTKVEI-K
```

Showing the sequence alignments of the final humanized versions of BB7 and DC1 kappa light chains. Gaps (-) are used to maintain Kabat numbering, and to show residue insertion or deletion where applicable.

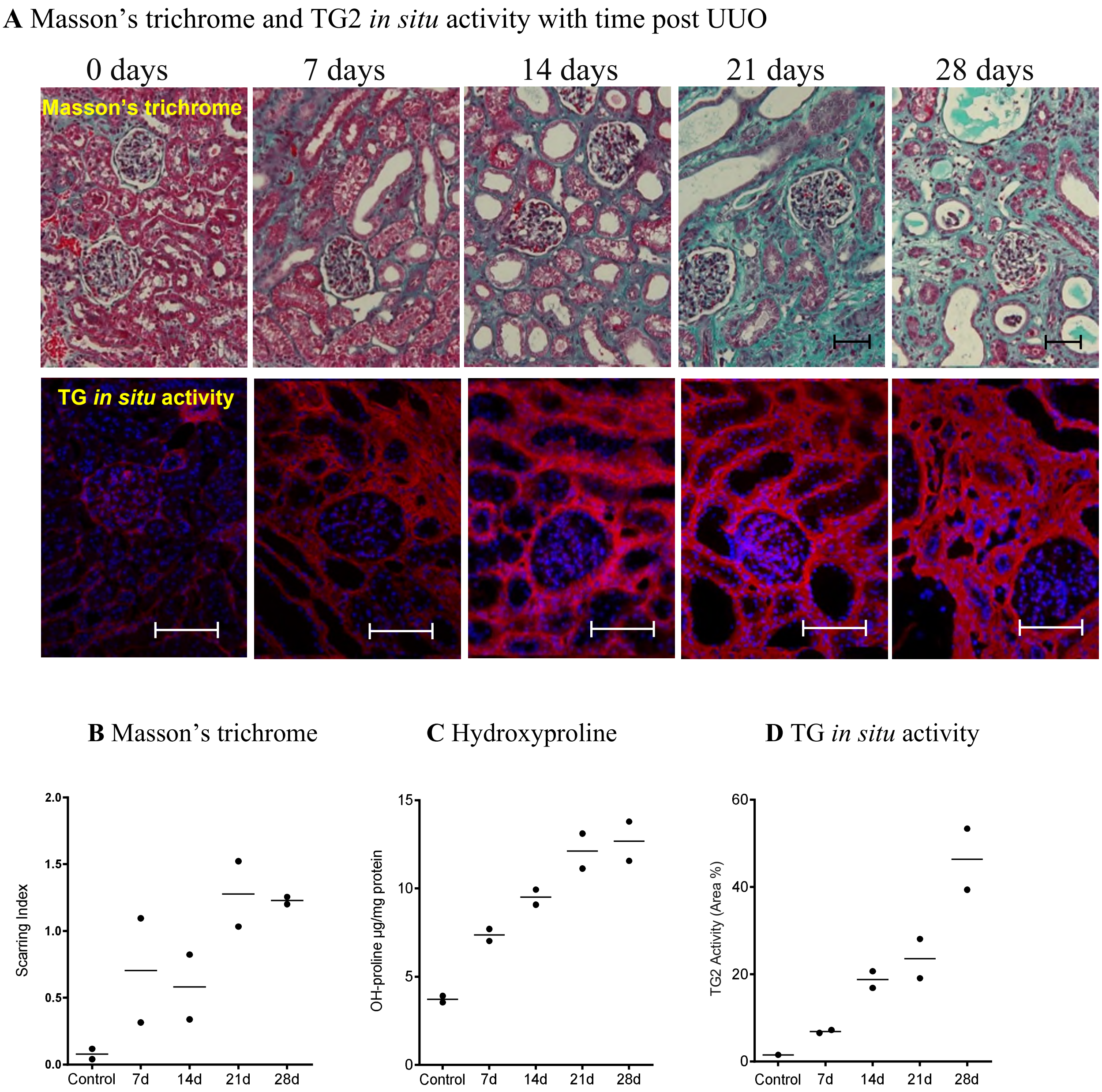

S1.1 Fig. Development of a UUO model in the New Zealand white rabbit. Eight New Zealand white rabbits underwent a left UUO with two animals culled every 7 days thereafter; the kidneys were recovered and analyzed for tubulointerstitial fibrosis and TG activity. Two additional animals received a sham operation. (A) Top row: Masson's trichrome staining showing an exemplar cortical image at x100 magnification from various time point. Blue staining indicates collagen; red/pink indicates cellular areas. Bottom row: corresponding images showing the level of TG activity at each time point. Red staining shows incorporation of TG substrate biotin cadaverine. (B) Quantitative computerized image analysis of whole

kidney scans of Masson's trichrome staining. Data represent mean scarring index ± SD. N = 2 per timepoint (0 days, n = 2; 7 days, n = 2; 14 days n = 2; 21 days, n = 2; 28 days, n = 2). (C) Quantification of total kidney collagen by amino acid analysis of hydroxyproline from acid-hydrolyzed kidney samples at each time point. Each data point signifies a mean value from the analysis of two independent kidney segments from each animal. Data represent mean ± SD hydroxyproline concentration per 20 µL of a 10% renal homogenate. n = 2 per group. (D) Quantification of TG2 activity by cadaverine incorporation on whole kidney scans. Activity was calculated using the proportion of orange versus blue staining across timepoints. Data represent mean TG activity ± SD. n = 2 per timepoint. Scale bar = 75 µm.

d, days; OH, hydroxy; SD, standard deviation; TG, transglutaminase; UUO, unilateral ureteral obstruction.

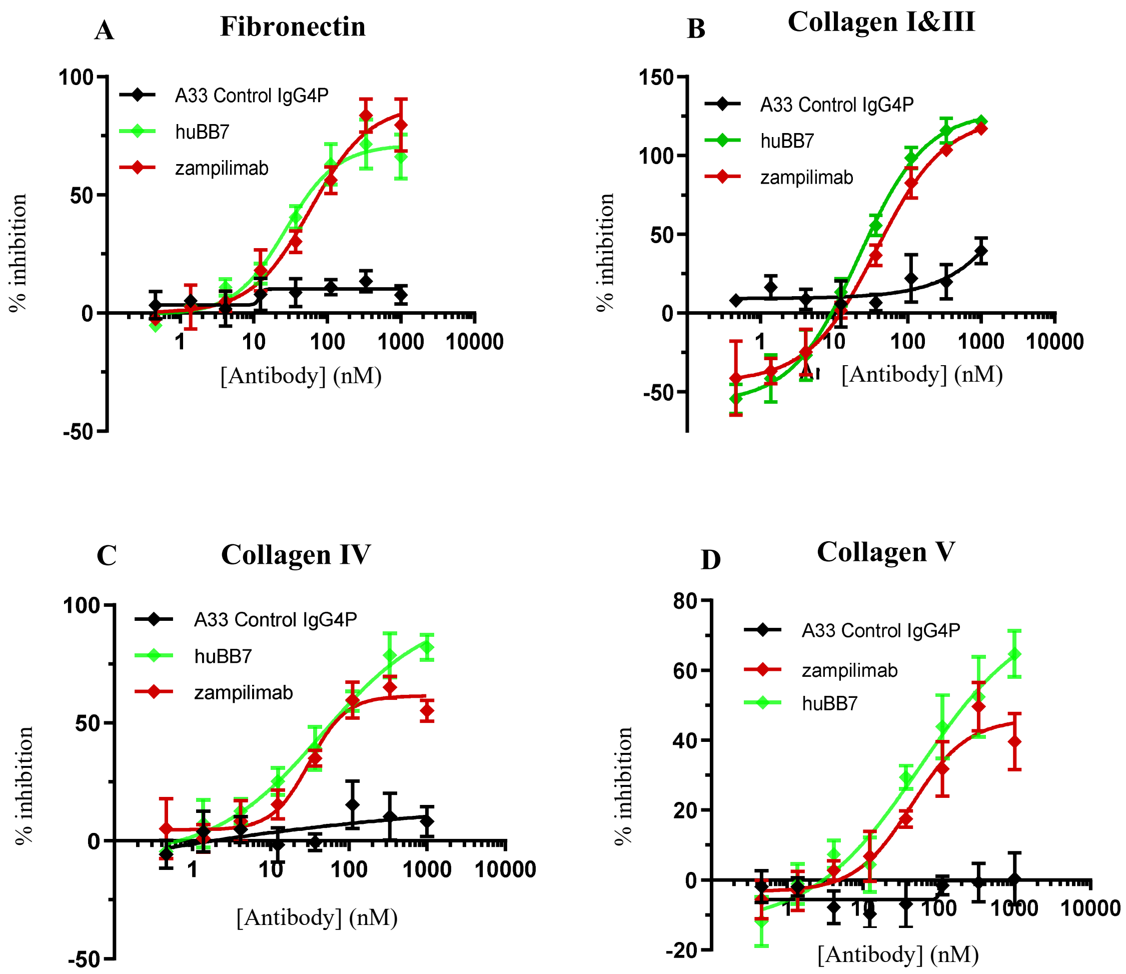

S1.2 Fig. ECM protein accumulation is inhibited by zampilimab in a primary human renal proximal tubule epithelial cell model of tubulointerstitial fibrosis: percentage inhibition. Percentage inhibition of the accumulation of (A) fibronectin, (B) collagen I and III, (C) collagen IV, and (D) collagen V in the ECM of RPTEC monocultures by zampilimab (red), huBB7 (green) or isotype control (A33 IgG4P; black) in cells induced with TGF- β1 (30 ng/mL). Calculated using the data in Fig 1B. Results are shown from one of four repeat experiments. Data represent mean level of percentage inhibition ± SD from eight technical repeats.

ECM, extracellular matrix; Ig, immunoglobulin; RPTEC, renal proximal tubule epithelial cells; TGF- β1, transforming growth factor-β1.

A Percentage inhibition

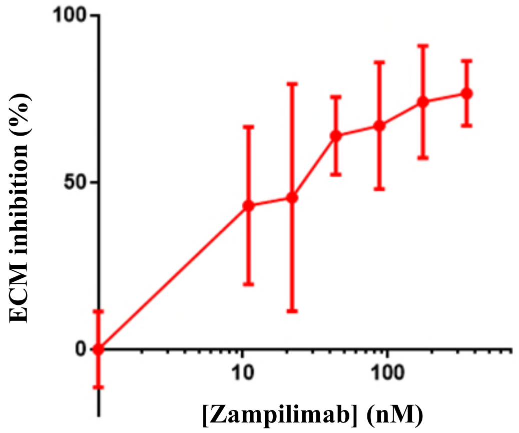

B Regression slope analysis

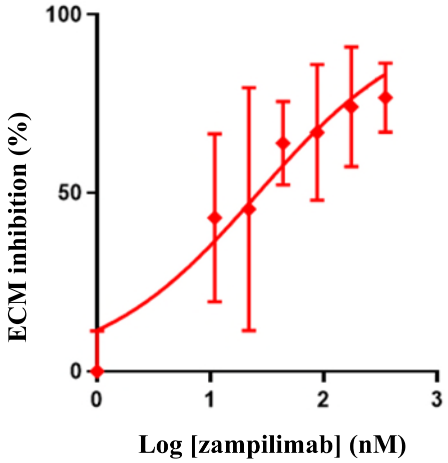

S1.3 Fig. TG2 inhibitory antibody zampilimab inhibits total ECM accumulation in a primary human renal proximal tubule epithelial cell model of tubulointerstitial fibrosis: percentage inhibition. Percentage inhibition of total ECM accumulation (measured using tritiated amino acid incorporation) by zampilimab (0–400 nM) in a primary human RPTEC model of tubulointerstitial fibrosis using induction with TGF-β1 (30 ng/mL). Data (Fig 1C) were calculated as a simple percentage inhibition (A) or log-transformed and a non-linear log (antagonist) versus response variable slope regression analysis (B). Data represent mean percentage inhibition (±SD).

ECM, extracellular matrix; RPTEC, renal proximal tubule epithelial cells; SD, standard deviation, TG2, transglutaminase 2; TGF-β1, transforming growth factor-β1.

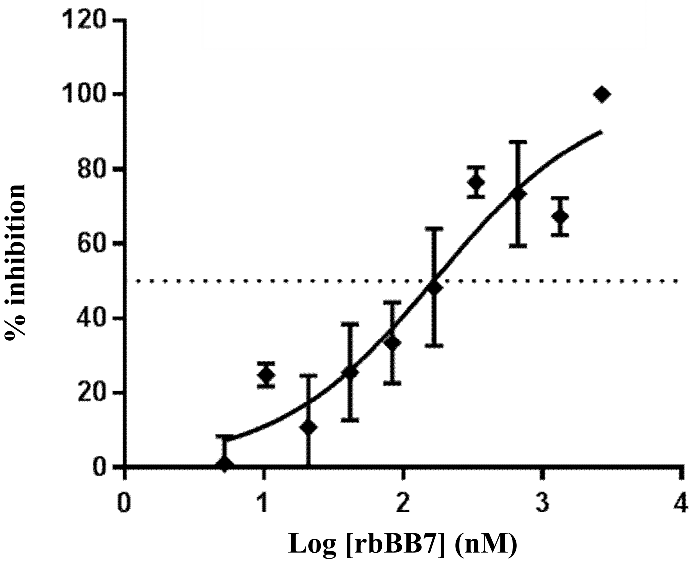

S1.4 Fig. TG2 inhibitory antibody rbBB7 inhibits total ECM accumulation in a primary rabbit renal proximal tubule epithelial cell and renal fibroblast co-culture model of tubulointerstitial fibrosis: percentage inhibition. Percentage inhibition of total ECM accumulation (as measured by isotopic ECM labelling) by rbBB7 (0.75–200 µg/mL) in a rabbit RPTEC and fibroblast co-culture model of tubulointerstitial fibrosis using induction of ECM by TGF-β1 (10 ng/mL). Data (Fig 2C) were log-transformed and a log (antagonist) versus response variable slope regression analysis performed. Data represent mean percentage inhibition (±SD).

ECM, extracellular matrix; RPTEC, renal proximal tubule epithelial cells; SD, standard deviation; TG2, transglutaminase 2; TGF-β1, transforming growth factor-β1.

A rbBB7 plasma levels in rabbit

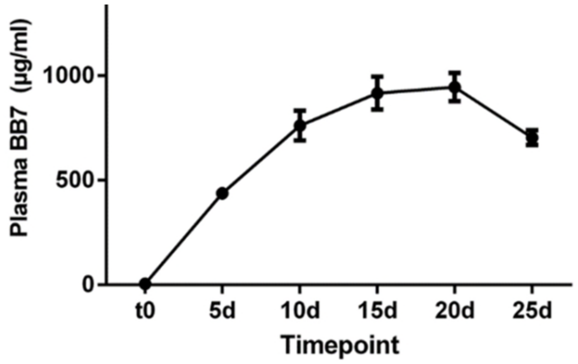

B Zampilimab plasma levels in cynomolgus monkey

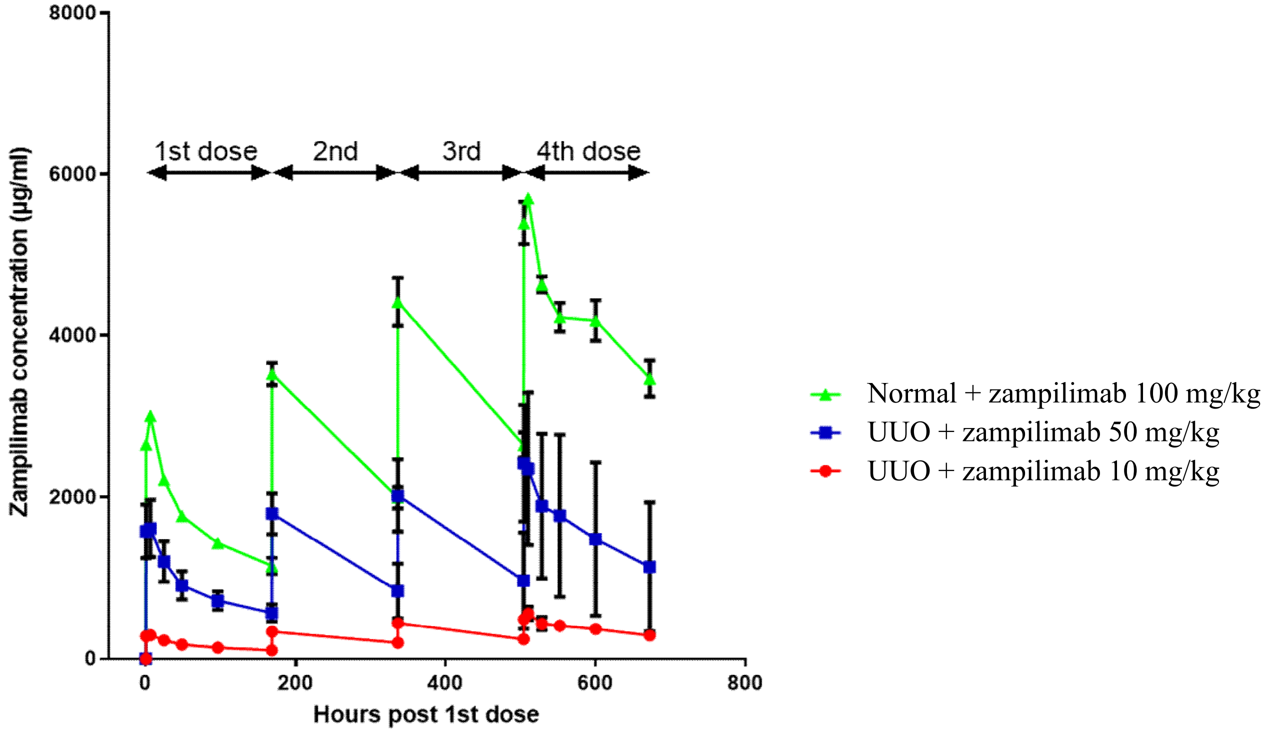

S1.5 Fig. Trough plasma concentrations of (A) rbBB7 in a rabbit UUO model and (B) zampilimab in a cynomolgus monkey UUO model show expected pharmacokinetics. Measurement of TG2 inhibitory antibodies rBB7 in a rabbit UUO study (Fig 4) and zampilimab in a cynomolgus monkey UUO (Fig 6) were made in plasma taken at trough (i.e., immediately before the next injection, which is equivalent to 1 IgG half-life) and in the cynomolgus monkey at additional time points. (A) Rabbit. Trough concentrations of rbBB7 in the UUO + rbBB7 group (n = 4) immediately before dosing at 5-day intervals. Antibody levels were measured by ELISA. The antibody signal at time 0 was below the threshold of

detection. Data represent mean plasma concentration of rbBB7 (±SD). (B) Cynomolgus monkey: plasma concentrations of zampilimab were measured by mass spectrometry in the UUO + zampilimab 10 mg/kg (n = 6) and UUO + zampilimab 50 mg/kg (n = 4) groups plus in a normal cynomolgus monkey treated with zampilimab 100 mg/kg (n = 2). Data represent mean plasma concentration of zampilimab (±SD).

d, day; ELISA, enzyme-linked immunosorbent assay; Ig, immunoglobulin; SD, standard deviation; TG2, transglutaminase 2; UUO, unilateral ureteral obstruction.

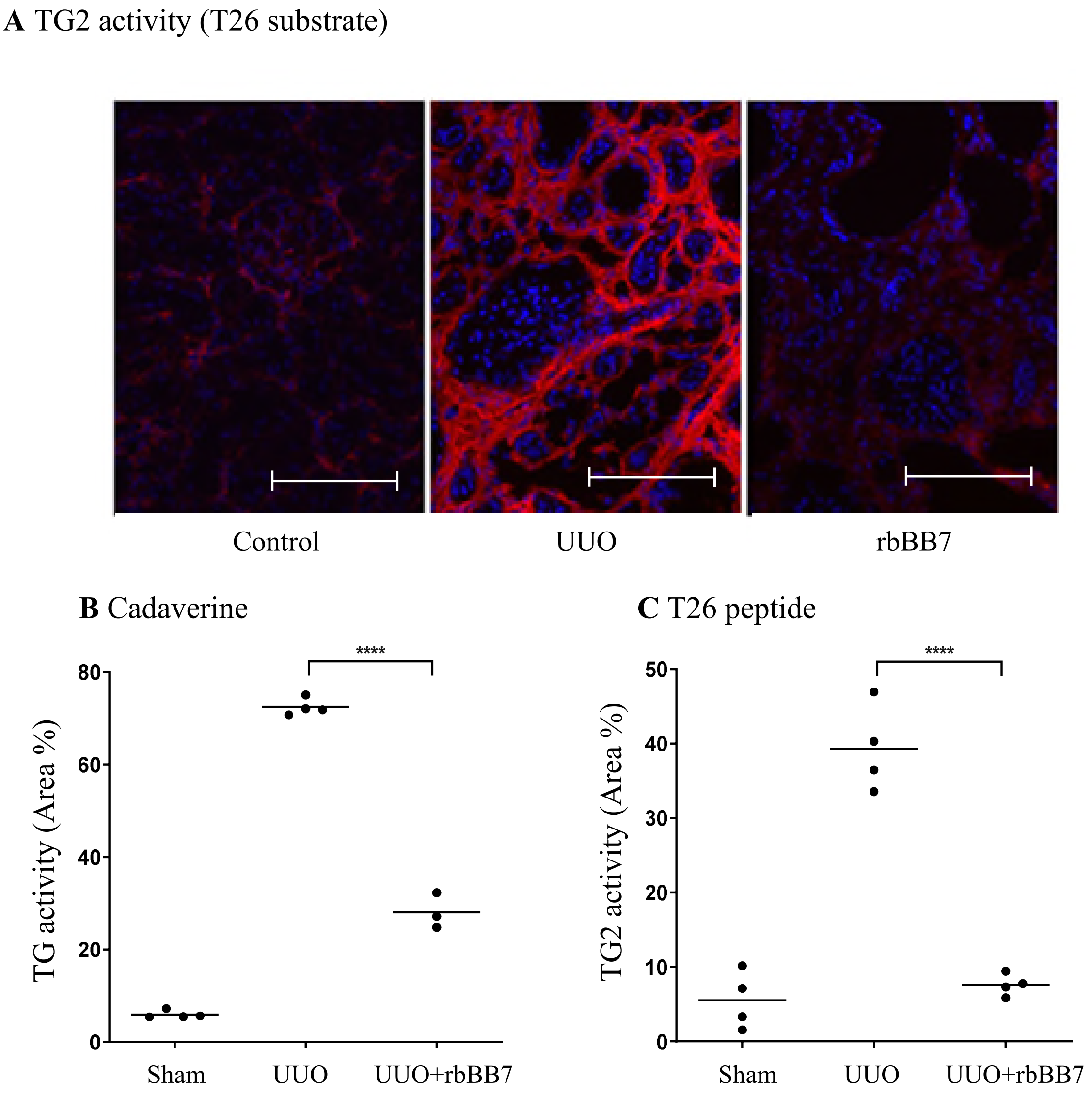

S1.6 Fig. rbBB7 is a potent inhibitor of in situ TG2 activity in a rabbit UUO model of CKD. Eight New Zealand white rabbits were subjected to a left UUO and kept for 25 days for tubulointerstitial fibrosis to develop**.** Four UUO animals received no treatment (UUO), and four received 100 mg/kg of TG2 inhibitory antibody rbBB7 (UUO + rbBB7) every 5 days. Four additional animals received a sham operation. The last dose of rbBB7 was given on Day 20 and measurements of TG activity were taken at termination (5 days, or one IgG half-life later). (A) Representative images showing staining of *in situ* TG2 activity measured by incorporation of the TG2 preferred substrate T26 peptide (red) in sections from the sham, UUO, and UUO + rbBB7 groups. Cell nuclei are shown in blue. Magnification

x200. (B) Quantification of total TG activity using the incorporation of the pan TG substrate cadaverine. Data represent mean percentage area of cadaverine incorporation ± SD. Significance was calculated by one-way ANOVA with Tukey's *post hoc* test. (C) Quantification of TG2 activity based on TG2 preferred substrate T26 peptide incorporation. Data represent mean percentage area of T26 incorporation ± SD. Significance was calculated by t-test. Scale bar = 75 μm.

****$p<0.00001$; ANOVA, analysis of variance; CKD, chronic kidney disease;
Ig, immunoglobulin; SD, standard deviation; TG, transglutaminase; UUO, unilateral ureteral obstruction.

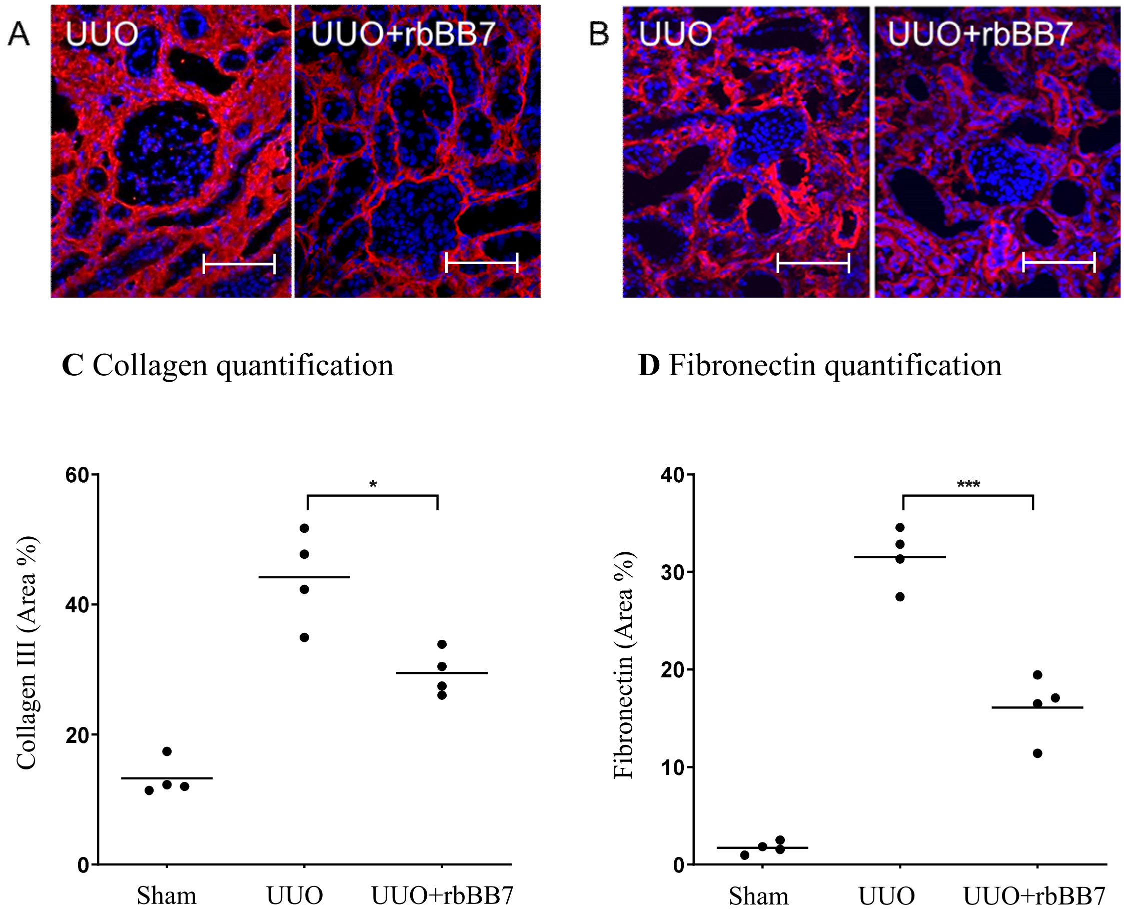

S1.7 Fig. rbBB7 prevents accumulation of collagen III and fibronectin in a rabbit UUO model of CKD. Eight New Zealand white rabbits were subjected to a left UUO and left for 25 days for tubulointerstitial fibrosis to develop. Four UUO animals received no treatment (UUO) and 4 received 100 mg/kg of TG2 inhibitory antibody rbBB7 (UUO + rbBB7) every 5 days. Four additional animals received a sham operation (sham). Collagen III (A) and fibronectin (B) were measured by immunofluorescence (red) on whole kidney sections, scanned, and the area of positive staining (as a percentage of the area of DAPI stain) measured by computerized high content image analysis using Definiens software. Magnification x200. (A&C) Exemplar images and quantification for collagen III. (B&D) Exemplar images and quantification for fibronectin. Data represent mean percentage area ± SD and significance shown by t-test. Scale bar = 75 µm.

*$p<0.01$; ***$p<0.0005$; CKD, chronic kidney disease; DAPI, 4′,6-Diamidino-2-phenylindole dihydrochloride; SD, standard deviation; TG2, transglutaminase 2; UUO, unilateral ureteral obstruction.

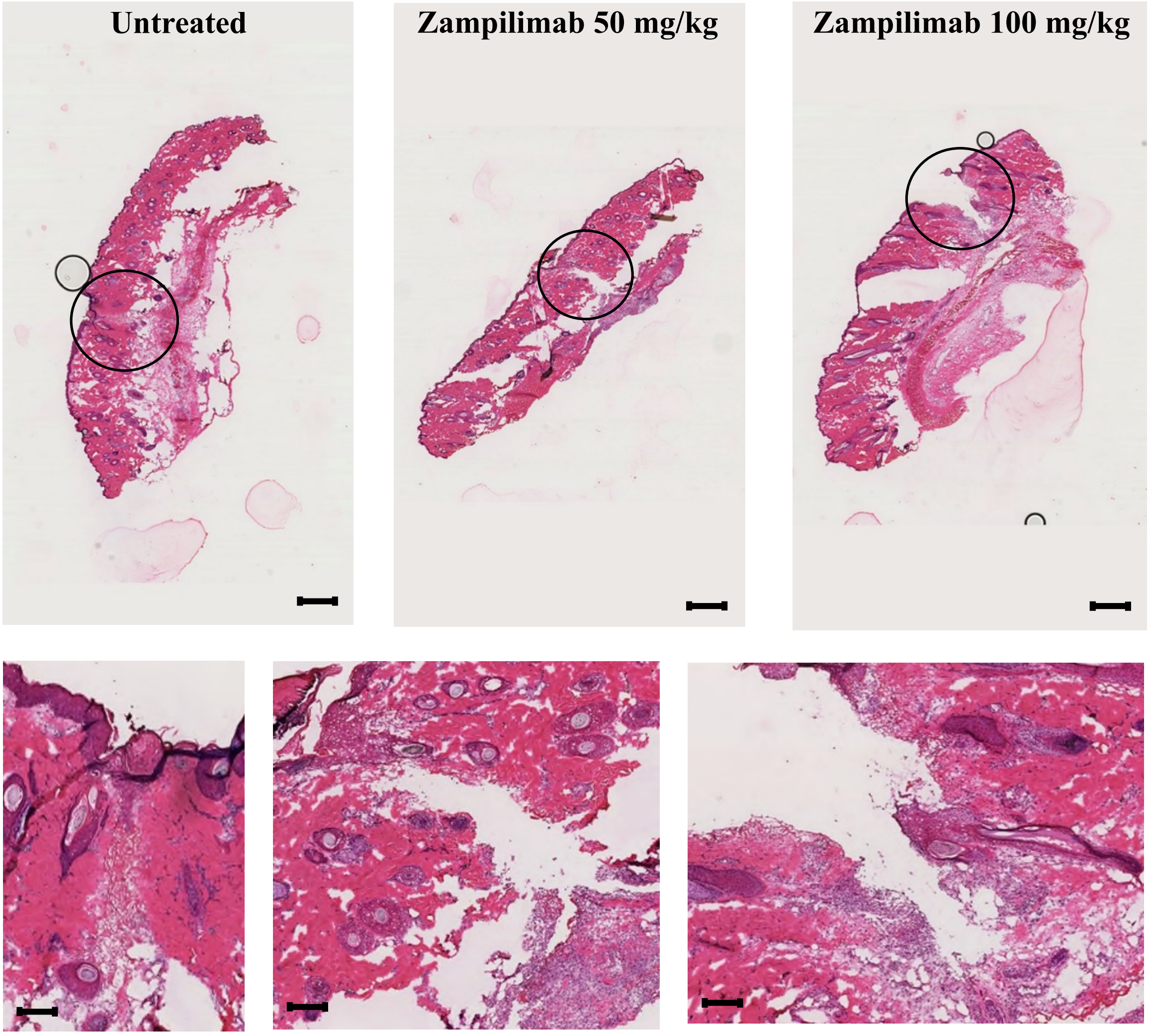

S1.8 Fig. Effect of zampilimab on dermal wound closure in cynomolgus monkeys.

Dermal wounds (6 per animal; 12 treated with zampilimab, 8 untreated) were made in the back skin of cynomolgus monkeys treated once weekly with up to 100 mg/kg IV zampilimab. Wounds were assessed up to 28 days for changes in closure and cell infiltration. Hematoxylin and eosin-stained images were obtained 3 days post injury from one untreated animal (left) and 2 zampilimab-treated animals (50 mg/kg [middle] and 100 mg/kg [right]). Circles indicate where the dermal wound is in the section. The left section (untreated) shows typical closure of the wound, the middle and right sections (zampilimab treated) show partial closure of the wound. Partial closure of a wound at 3 days, as exemplified in the treated animals, was equally prevalent in untreated and zampilimab-treated animals and is within the natural

variation of early wound closure. Of the wounds examined at 3 days post injury (6 per animal), no clear alteration in wound closure was observed, with typically 2 out of 6 wounds remaining partly open irrespective of treatment. No open wounds were observed after 3 days in any animal. Overall, no clear differences in wound healing between control and zampilimab-treated animals were reported by the pathologist. Scale bar ~1000 µm (top row, low power); ~50 µm (bottom row, high power). IV, intravenous.

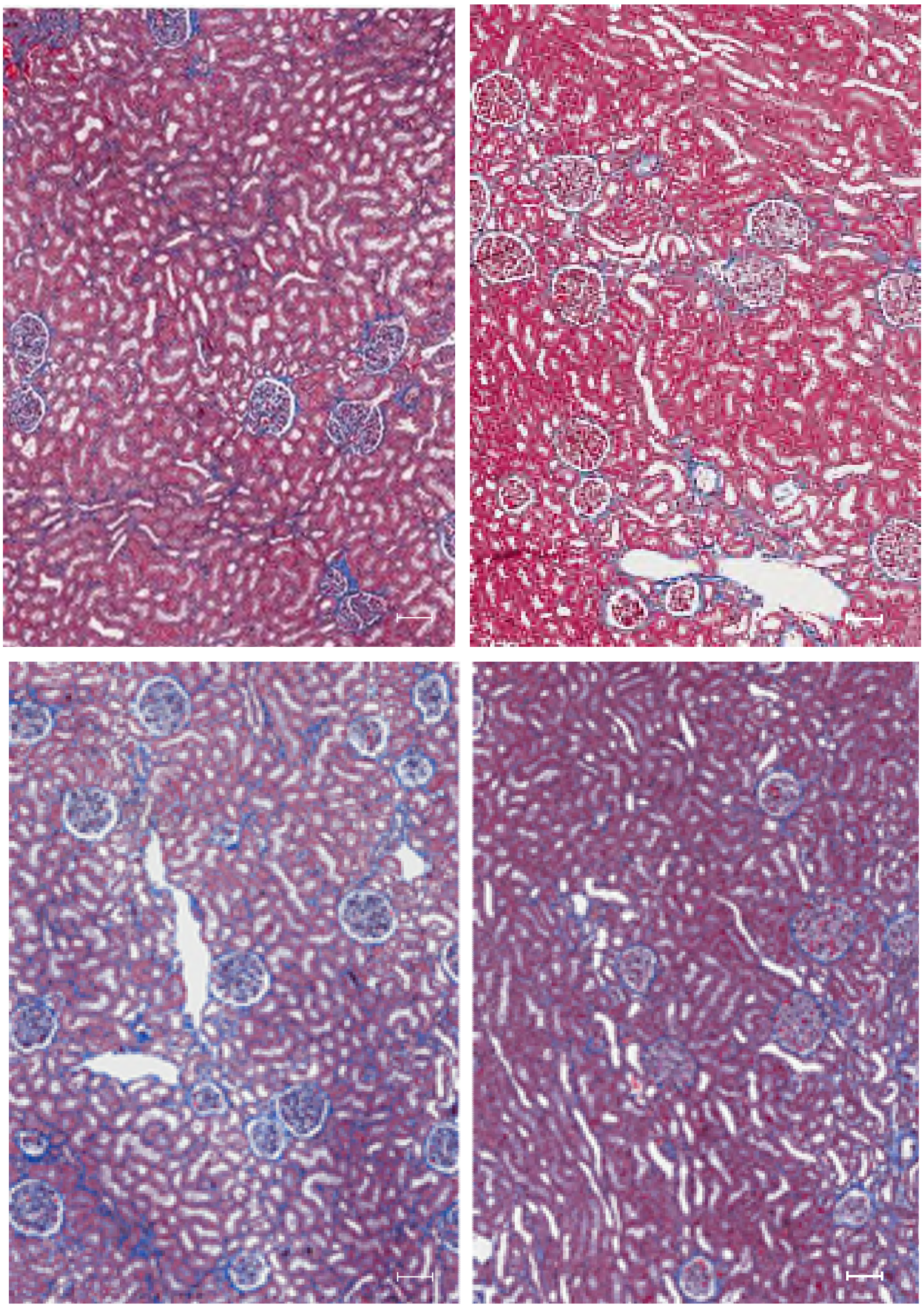

Normal

Normal
+ zampilimab 100 mg/kg

S1.9 Fig. Zampilimab does not affect normal kidney histology in cynomolgus monkeys. Kidneys from 2 normal cynomolgus monkeys (left) and 2 normal cynomolgus monkeys treated for 28 days with zampilimab 100 mg/kg (right) were sectioned, stained with Masson's Trichrome and examined microscopically. 100 x magnification. Scale bar = 75 µm.

Part S2: Supplemental toxicology and pathology information

Methods: Toxicology

Hematology and clinical chemistry

Blood (6 mL) was collected for hematology and clinical chemistry before surgery (including sham operations) and again during the last week before termination. One mL blood was collected in EDTA tubes for hematology analysis and the remainder was collected into a plain tube to produce serum for clinical chemistry.

Hematology

The parameters detailed in S2.1 Table (below) were determined using an automatic blood analyzer (SYSMEX XE2100).

S2.1 Table. Hematology parameters measured.

**Parameter**	**Abbreviation**	**Parameter**	**Abbreviation**
White blood cell	WBC	Mean corpuscular hemoglobin	MCH
Neutrophil	NEU	Mean corpuscular hemoglobin concentration	MCHC
Lymphocyte	LYMPH	Red cell distribution width	RDW
Monocyte	MONO	Platelet	PLT

Eosinophil	EO	Platelet large cell ratio	P-LCR
Basophil	BASO	Mean platelet volume	MPV
Red blood cell	RBC	Platelet distribution width	PDW
Hemoglobin	HGB	Plateletcrit	PCT
Hematocrit	HCT	Reticulocyte	RET
Mean corpuscular volume	MCV		

Clinical chemistry on serum

The parameters presented in S2.2 Table (below) were determined using automatic blood chemistry analyzer (OLYMPUS AU5400).

S2.2 Methods Table. Clinical chemistry parameters measured in serum.

**Parameter**	**Abbreviation**	**Parameter**	**Abbreviation**
Alanine aminotransferase	ALT	Glucose	GLU
Aspartate aminotransferase	AST	Creatinine	CREA
Alkaline phosphatase 2S	ALP2S	Total bilirubin	TBILI
Albumin	ALB	Calcium	Ca^{2+}
Globulin	GLB	Magnesium	Mg^{2+}

Albumin/Globulin ratio	A/G	Sodium	Na^+
Creatine kinase	CK	Potassium	K^+
Total cholesterol	TC	Chloride	Cl^-
Total protein	TPROT	Inorganic phosphorus	P

Urinalysis

During the early morning, urine samples were collected by putting monkeys in a metabolic cage for 2 hours, once during the pre-test phase and once during the last week of treatment. Volume and density were recorded; 2 mL were used for semi-quantitative determinations (including protein and glucose as a minimum) by using multistrip, followed by dry chemistry analysis (to compare the color of the different samples]). All determinations presented in S2.3 Methods Table (below) were conducted on fresh samples.

S2.3 Methods table. Urine analysis parameters.

**Parameter**	**Abbreviation**	**Parameter**
Urobilinogen	URO	pH
Bilirubin	BIL	Color
Ketone bodies	KET	Clarity
Blood	BLD	Cast

Protein	PRO	
Nitrite	NIT	
White blood cell	WBC	
Glucose	GLU	
Specific gravity	SG	
Red blood cell	RBC	
Epithelial cell	EC	

Methods: Pathology

Necropsy and tissue preservation

A full macroscopic examination of the collected tissues was performed on animals by the sitting veterinarian under the general supervision of a pathologist, and all lesions were recorded.

Histology/pathology

At study termination, animals were sacrificed by phenobarbital overdose followed by exsanguination. All tissues listed in S2.4 Methods Table (below) were collected, macroscopically examined, and fixed in 10% neutral-buffered formalin. Tissues marked with an X in the column “tissue examined” were embedded in paraffin wax BP (block stage), sectioned at a nominal 5 µm, and stained with hematoxylin and eosin (H&E). These were subsequently microscopically examined by a study pathologist and any abnormality reported.

Blood smear and bone marrow smear (Groups 1, 2, 4, 5): blood smear and bone marrow smear (sternum) were prepared at termination. Slides were fixed and stained with Wright-Giemsa and examined by the pathologist. A detailed sample collection/preparation procedure is given in the pathology report.

S2.4 Methods Table. Tissue list.

**Tissue/organ preserved**	**Organ weighed**	**Tissue examined**	**Tissue/organ preserved**	**Organ weighed**	**Tissue examined**
Adrenals	X		Nares		
Animal identification			Nasopharynx		
Aorta			Esophagus		
Bone marrow smear (femur)		X	Optic nerves		
			Ovaries		
Brain	X	X	Oviducts		
Cecum		X	Pancreas		X
Colon		X	Peyer's patch		
Dosing sites		X	Pituitary		
Duodenum			Prostate		
Eyes			Rectum		
Femur with bone marrow and stifle joint			Salivary glands – mandibular, sublingual, parotid		
Gall bladder			Sciatic nerves		
Gross lesions		X	Seminal vesicles		
Harderian glands			Skin and subcutaneous tissue		X
Head (not processed)					

**Tissue/organ preserved**	**Organ weighed**	**Tissue examined**	**Tissue/organ preserved**	**Organ weighed**	**Tissue examined**
Heart	X	X	Spinal cord (cervical, thoracic, lumbar)		
Ileum		X	Spleen	X	X
Jejunum			Sternum with bone marrow		
Kidneys	X	X	Stomach		
Lacrimal glands			Testes with epididymides		
Larynx			Thymus	X	X
Liver	X	X	Thyroid (with parathyroid)		
Lungs with mainstem bronchi and bronchioles		X	Tongue		
			Trachea		
Lymph node – mandibular			Trachea bifurcation		
Lymph node – mesenteric			Ureters		
Lymph node – popliteal		X	Urinary bladder		X
Mammary area			Uterus including cervix		
Muscle (quadriceps)			Vagina		
Nasal cavity			Zymbal glands		

Results: Toxicology and pathology

Raw toxicology and pathology data

The raw toxicology and pathology data are referred to here in the Supplemental Results section and presented in full in the Supplemental Excel file (S2.6 to S2.45 Tables) as follows:

- Clinical Chemistry (for selection) (S2.6 Table)
- Histology (S2.7 Table)
- Weight (S2.8 to S2.9 Tables)
- Clinical Chemistry (S2.10 to S2.16 Tables)
- Complete Cell Counts (S2.17 to S2.28 Tables)
- Urinalysis (S2.29 to S2.38 Tables)
- Bone Marrow (S2.39 to S2.44 Tables)
- Blood smear (S2.45 Table).

Microscopic examination of tissue samples

Selected tissue samples, including brain, skin, thymus, urinary bladder, heart, popliteal lymph node, liver, ileum, cecum, colon, spleen, lung, kidney, pancreas and the injection site, were collected and macroscopically examined; no apparent abnormalities were observed. All tissue slides were examined microscopically (see S2.8 Table in the Weight tab of the Supplemental Excel file).

The right (non-UUO) kidneys of all animals showed normal morphology apart from one animal that received zampilimab 50 mg/kg (C0901373) and showed minimal tubular dilation.

No histopathological changes were noted in the examined tissues/organs including kidneys in the sham + formulation buffer group.

The two animals in the normal + zampilimab 100 mg/kg group showed increased centrilobular glycogen vacuoles (left kidney). In addition, one animal presented bilateral tubular dilation in the renal cortex. The relationship of this change with administration of zampilimab is uncertain given the low number of animals in this group.

No macroscopic change was observed at the injection site of any animal; the only microscopic changes observed were minimal and mild hemorrhage and inflammatory cell infiltration, which are commonly observed with the subcutaneous administration of therapeutic antibodies. Due to inadequate tissue sampling in several animals, an accurate evaluation of this tissue was not possible.

All other microscopic findings were considered spontaneous changes in Cynomolgus monkeys of this age (see S2.8 Table in the Weight tab of the Supplemental Excel file).

Clinical pathology

Blood samples were collected before surgery and at termination (4 weeks later), for determination of hematology and clinical chemistry. Data showed some elevated ALT and AST values, out of the normal range during pre-test or at the end of the study but without correlation with UUO surgery or zampilimab treatment (S2.10 Table in the Clinical Chemistry tab of the Supplemental Excel file). Although albumin levels were within the normal range, there was a trend toward decreased levels at study termination in UUO animals and UUO animals treated with zampilimab 10 mg/kg. The decrease was not observed with the 50 mg/kg dose, suggesting a protective effect of the highest dose of zampilimab (S2.11 Table in the Clinical Chemistry tab of the Supplemental Excel file).

Urea and creatinine levels were increased 4 weeks after UUO surgery in the presence or in the absence of zampilimab (S2.13 Table in the Clinical Chemistry tab of the Supplemental

Excel file). By contrast, no significant changes were observed in animals undergoing no ligation. Other parameters were within control ranges and no real effect of UUO or zampilimab treatment could be evidenced.

Hematology data indicated no significant effects of UUO surgery and/or zampilimab treatment. The reticulocyte count was elevated in all groups at the end of the study, without clear effect of the UUO surgery and/or the treatment with zampilimab (S2.17 to S2.28 Tables in the Complete Cell Counts tab of the Supplemental Excel file).

Urine analyses performed before and after surgery indicated mainly no changes in bilirubin, urobilinogen, ketones, proteins, nitrites determined by dipstick, no changes in pH, color or microscopic examination of sediments. However, blood score (by dipstick) was higher for animals with UUO, with or without zampilimab 10 mg/kg, suggesting kidney damage after UUO and some protection with the 50 mg/kg dose of zampilimab (Supplemental Fig 2.1 below and S2.29 to S2.38 Tables in the Urinalysis tab of the Supplemental Excel file).

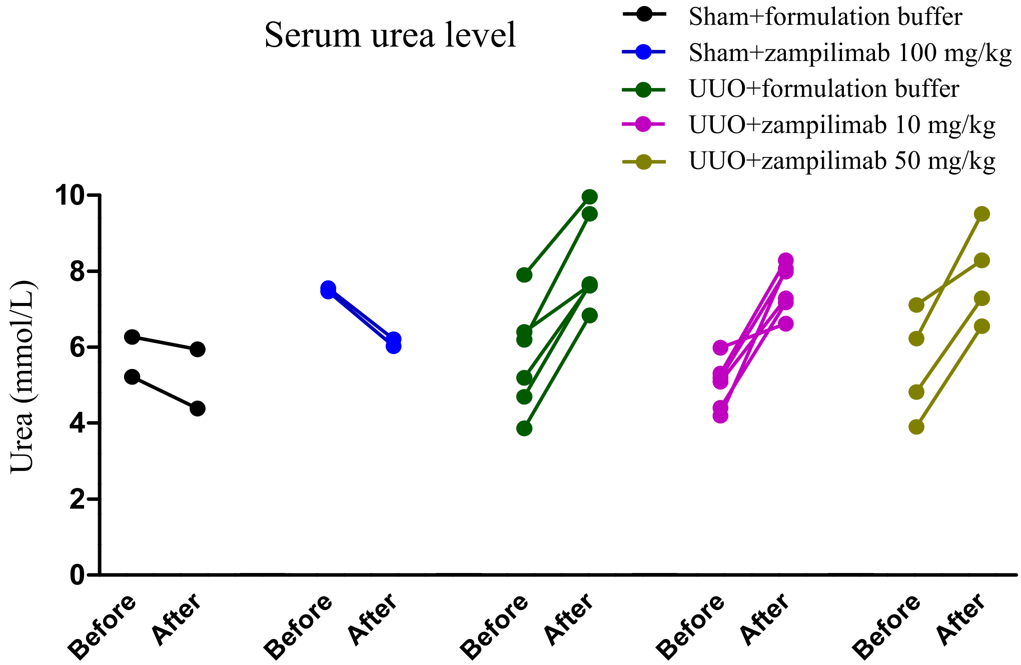
Serum urea level
Sham+formulation buffer
Sham+zampilimab 100 mg/kg
UUO+formulation buffer
UUO+zampilimab 10 mg/kg
UUO+zampilimab 50 mg/kg
Urea (mmol/L)
0
2
4
6
8
10
Before
After
Before
After
Before
After
Before
After
Before
After

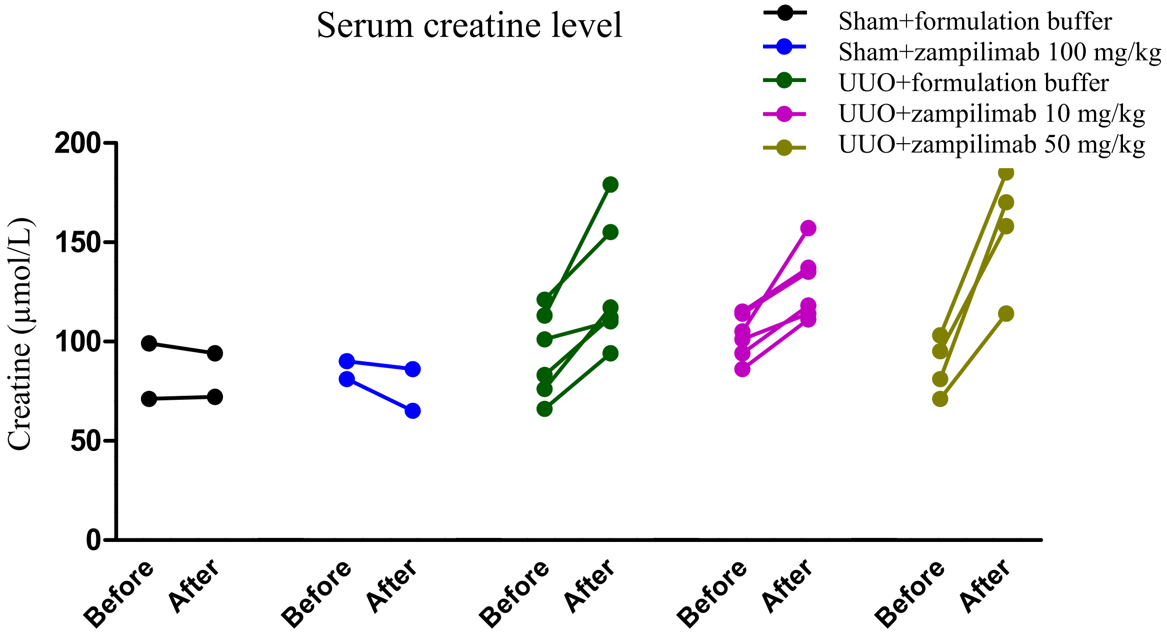
Serum creatine level
Sham+formulation buffer
Sham+zampilimab 100 mg/kg
UUO+formulation buffer
UUO+zampilimab 10 mg/kg
UUO+zampilimab 50 mg/kg
Creatine (µmol/L)
0
50
100
150
200
Before
After
Before
After
Before
After
Before
After
Before
After

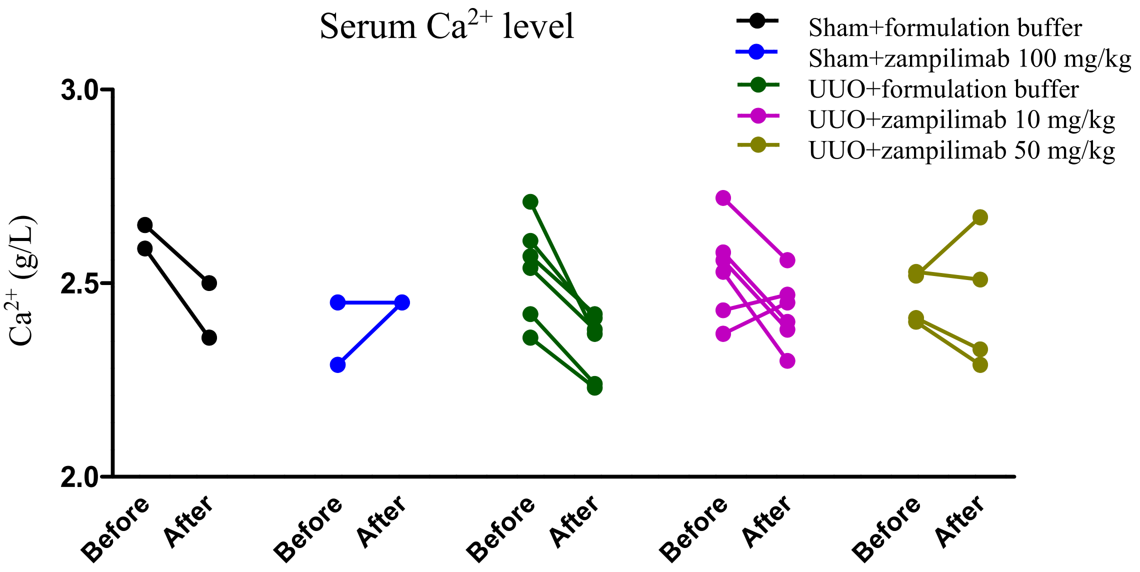

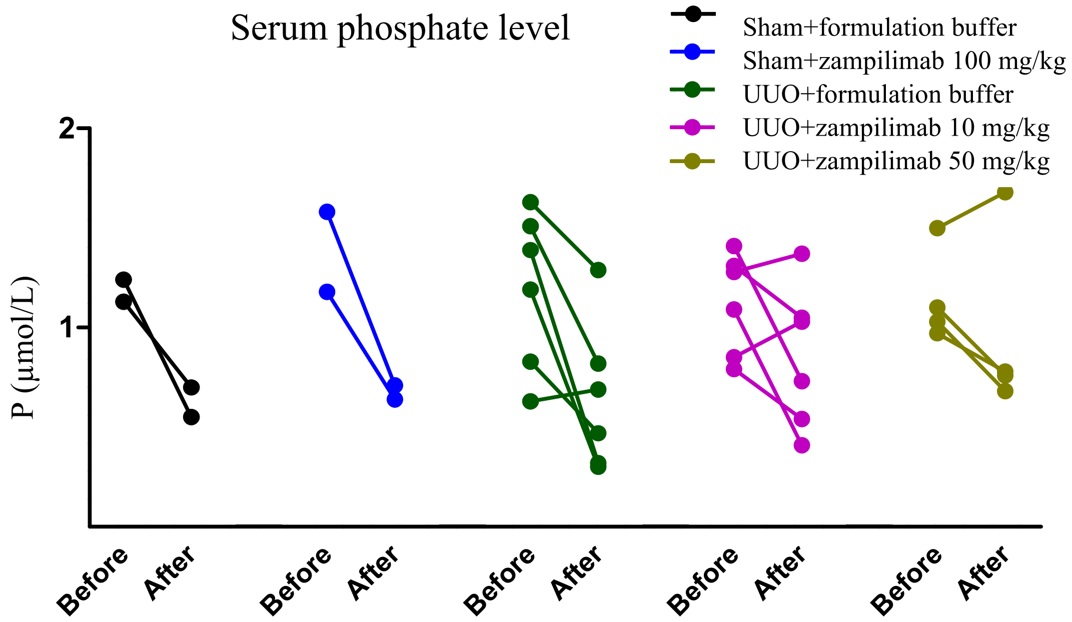

S2.1 Fig. Effect of UUO and weekly zampilimab treatment for 4 weeks on serum levels of urea, creatinine, calcium, and phosphate. Blood samples (Groups 1, 2, 4 and 5) were collected before (Baseline) and 4 weeks after UUO (at termination), serum were prepared and analyzed.

$Ca^{2+,}$ calcium ion; P, phosphate; UUO, unilateral ureteral obstruction.

Bone marrow and blood smear examination

Bone marrow and blood smears were prepared from samples collected at termination (S2.45 Table) and examined by a hematologist.

S2.5 Table. Animal list for bone marrow and blood smear analysis.

Group and Treatment	Animal No.
UUO + formulation buffer	C0704039, C0812509, C0801821, C0806567, C0803553, C0910081
UUO + zampilimab 10 mg/kg	C0805575, C0808537, C0810515, C0804557, C0804531, C0902313
UUO + zampilimab 50 mg/kg	C0807539, C0901373, C0810517, C0810519
Sham + formulation buffer	C0809035, C0903123
No operation + zampilimab 100 mg/kg	C0812507, C0902195

The data showed that: all of the slides had low numbers of fat droplets, occasional spicules and megakaryocytes were also seen. No abnormalities were noted at low magnification (S2.38 to S2.44 Tables) in the Bone Marrow tab of the Supplemental Excel file).

Three animals (C0704039 from UUO + formulation buffer; C0808537 and C0810515 from UUO + zampilimab 10 mg/kg) had high myeloid aggregate and high myeloid:erythroid

(M:E) ratio. These were marginally above the maximal value seen in control animals. The significance as to whether these findings reflect an effect of UUO surgery remains uncertain. No morphological abnormalities were associated with this finding and no other cell types were altered in number.

All other animals had myeloid cells that were morphologically normal and in proportionate pyramidal numbers.

There were no morphological abnormalities found in erythroid line and all animals had cell numbers that reflected an orderly maturation sequence.

No abnormalities were found with lymphoplasmacytic cells and other cell types.

No abnormalities were found on blood smears (S2.45 Table in the Blood Smear tab of the Supplemental Excel file).

Conclusion

UUO animals dosed with zampilimab at 10 or 50 mg/kg/week for 4 weeks and control animals dosed with zampilimab at 100 mg/kg/week for 4 weeks did not show evidence of toxicity based on hematology, pathological examination, blood chemistry and urinalysis.
